# A reliable starfish optimization algorithm for establishing equivalent circuit of proton exchange membrane fuel cell

**DOI:** 10.1038/s41598-026-54194-9

**Published:** 2026-06-13

**Authors:** Mohamed Hashem, Hossam Hassan Ali, Ahmed Fathy, Mohamed Khamies

**Affiliations:** 1 Department of Electrical Engineering, Faculty of Engineering, New Valley University, New Valley, 72511 Egypt; 2 Electrical Department, Faculty of Technology and Education, Sohag University, Sohag, 82524 Egypt; 3 Department of Electrical Engineering, College of Engineering, Jouf University, 72388 Sakaka, Saudi Arabia; 4 Department of Electrical Engineering, Faculty of Engineering, Sohag University, Sohag, 82524 Egypt

**Keywords:** Starfish optimization algorithm, Parameter estimation, Optimization, PEMFC

## Abstract

The proton exchange membrane fuel cell (PEMFC) can be effectively simulated, managed, and controlled via accurate identification of its model’s unknown parameters. The nonlinear properties of fuel cells (FCs) and the absence of complete data in the datasheet cause certain obstacles in this procedure. This study suggests a novel approach employing the starfish optimization algorithm (SFOA) for designing the equivalent circuit of PEMFC by evaluating its parameters utilizing experimental data. This algorithm is highly efficient as well as avoiding local optima due to exploitation and exploration balance. Five distinct FCs are analyzed: Horizon_H12, Horizon H-1000XP PEMFC stack, Temasek_1kw, SR-12PEM 500-W, and BCS-500W PEMFC stacks. The suggested SFOA is evaluated in comparison with previously published techniques of seagull optimization algorithm (SOA), escape optimizer (ESC), gold rush optimizer (GRO), tunicate swarm algorithm (TSA), beluga whale optimization (BWO), success history intelligent optimizer (SHIO), enhanced transient search optimizer (ESTO) and Newton–Raphson-based optimizer (NRBO). Decreasing the sum square error (SSE) between the calculated and measured output voltages is the targeted goal. The minimum values of SSE obtained through the suggested SFOA are 4.662696E−04, 3.948527E−01, 5.4322160E−01, 1.056370, and 1.169778E−02 for Horizon_H12, Horizon H-1000XP PEMFC stack, Temasek_1kw, SR-12PEM 500-W, and BCS-500W PEMFC stacks, respectively. Additionally, the PEMFC dynamic simulation model is constructed in Matlab/Simulink, while step load disruption is employed for analyzing the model’s performance. The acquired results indicate the success of the suggested SFOA in constructing dependable models of different PEMFCs.

## Introduction

### Background

**Fig. 1:**
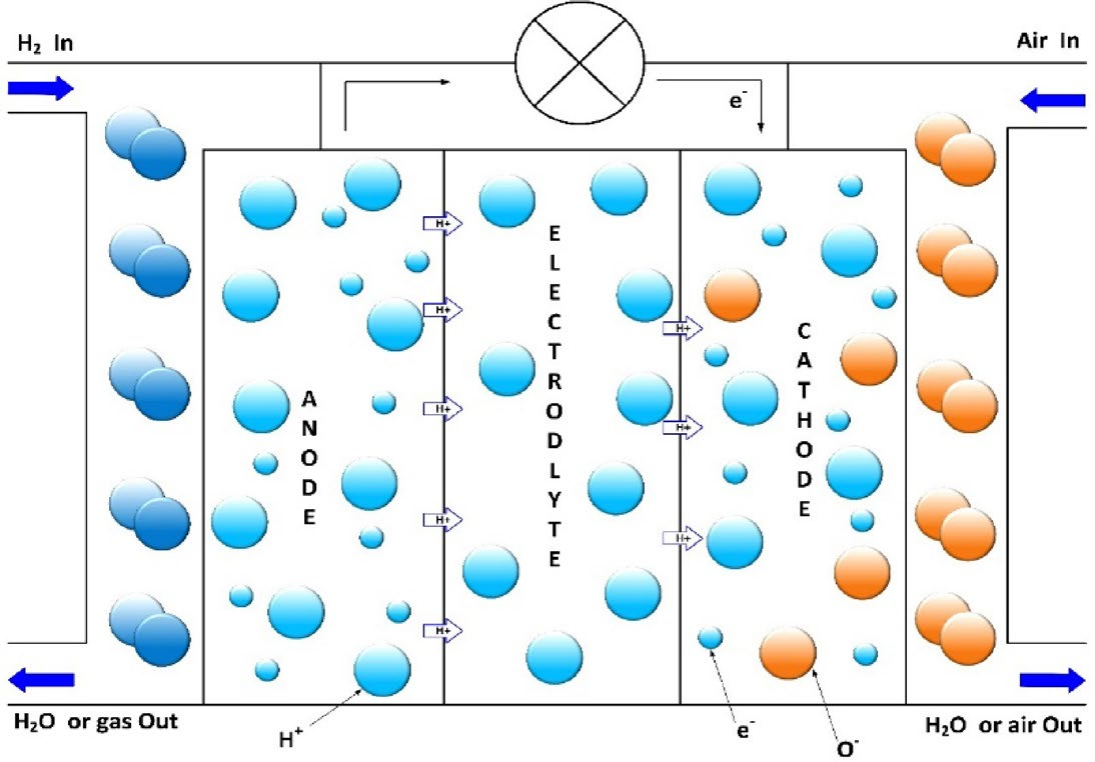
Schematic diagram for the PEMFC’s electrochemical operation.

Recently, the clean renewable energy requirement has become crucial in the electrical systems all over the world due to the global warming issues resulting from fossil fuel sources. The fast development of renewable technology is increased as the energy consumption’s growth brought on by new industrial works and population has a widespread range of applications^1,2^. Among these distinguished sources is fuel cells (FCs), which are widely employed as a clean and reliable energy source; they depend on the electrochemical responses between oxygen and hydrogen for producing the electrical energy from chemical energy. They are characterized distinct merits including (i) no moving parts, (ii) high efficiency and robustness, (iii) minimum operation cost, (iv) no power emission, and (v) friendly environmental^3,4^. FCs are applied in various applications including, electric vehicles (EVs), shipping industry, mobile phone recharging, and providing energy for residential and commercial buildings^5^. The FCs kinds are categorized depending on the electrolyte type and start-up time needed. On the other hand, the FC’s temperature, pressure, humidity, and stack efficiency have significant impacts on its technical performance^6^. Among the numerous kinds of FCs, the proton exchange membrane fuel cell (PEMFC) represents one of the most attractive types implemented in many applications like portable power sources and stationary applications. This is because it has excellent advantages involving (i) great power density, (ii) great efficiency, (iii) low operating temperature, (iv) fast response, (v) best reliability, and (vi) appropriate volume and weight^7–10^. It is a complex nonlinear system which consists of different parts such as (i) hydrogen and air supply systems, (ii) cooling and control systems, as well as (iii) PEMFC stacks^11^. Figure 1 depicts the operating principle of the PEMFC system^12^. With the aid of a catalyst and hydrogen, the primary reactant is delivered to the PEMFC’s anode, where it experiences an oxidation–reduction reaction to create hydrogen ions and electrons. Water is created when hydrogen ions mix with oxygen atoms from the surrounding air at the cathode after flowing through the fuel membrane. A load current is formed when electrons flow through an external circuit in order to attain the cathode because they are unable to pass through the electrolyte. Water and surplus gases are the only outputs of this procedure^13,14^.

Improving the PEMFC efficacy and efficiency has emerged as a crucial research topic. A key component of this work is modeling PEMFC operation. The empirical model with unknown parameters, which is extensively employed and less complex than the mechanical model, defines the cells’ external characteristics using empirical equations derived from experiments. On the other hand, the mechanical model utilizes chemical reaction equations and the rules of mass and heat conservation to explain the cells’ internal information. Calculating the model’s parameters is still significantly challenging due to the absence of the PEMFCs’ nonlinear characteristics. The inherent variability and uncertainty of these parameters lead to a difficult task for their extraction, which is required for the operation and control of the model^15^. Therefore, talking about the problem of PEMFC models’ parameters assessment is a must.

### Literature review

Various studies concerned with the PEMFC parameters assessment have been demonstrated using many optimization techniques, they can be divided into conventional techniques and meta-heuristic approaches. The first one includes impedance characterization^16^, the current change method^17^, fractional-order modeling^18^, gradient-based restoration techniques (GRG)^19^, the electric bicycle system^20^, and impedance spectroscopy^21^. However, these conventional techniques have significant restrictions in effectively evaluating model parameters due to the multi-variable complexity and the extremely non-linear characteristics of the PEMFC systems^22^.

On the other hand, the second classification represents the meta-heuristic optimization techniques that have been developed for addressing the weakness associated with conventional methods. This can be achieved through dealing with the extremely non-linear optimization problems, such as the PEMFCs’ nonlinear characteristics, and achieving the best solutions associated with minimum computational burden and great accuracy. Hence, various research studies used the meta-heuristic approaches to estimate and optimize the undefined parameters of PEMFC by mitigating the sum of square error (SSE) or the mean absolute error (MAE) or the mean square error (MSE) between the measured cell voltage and the evaluated voltage across a given dataset^23^. These optimization techniques involve well-established algorithms such as chaotic harris hawks optimizer^24^, tree growth algorithm^25^, moth-flame optimizer^26^, hybrid vortex search algorithm and differential evolution^27^, modified artificial ecosystem optimizer^28^, coyote algorithm^29^, pathfinder algorithm^30^, slime mould algorithm^31^, improved Archimedes optimizer^32^, harris hawks’ optimization and atom search optimization algorithms^33^, chaos-embedded particle swarm^34^, equilibrium optimizer^35^, heap-based optimization algorithm^36^, modified harris hawks optimizer^37^, improved chaotic mayfly optimization algorithm^38^, heterogeneous comprehensive learning Archimedes optimizer^39^, gradient-based optimizer^40^, bald eagle search optimizer^41^, enhanced transient search optimizer^42^, enhanced bald eagle algorithm^43^, improved evaporation rate water cycle algorithm^44^, improved gorilla troops algorithm^45^, chaotically based-bonobo optimizer^46^, swarm intelligence algorithm^47^, hybrid osprey algorithm and the coati algorithm^48^, war strategy algorithm^49^, Kepler optimization algorithm^50^, social learning-based optimization^51^, spotted hyena optimizer^52^, multi-strategy tuna swarm optimizer^53^, dynamic ant colony optimizer^54^, enhanced salp swarm algorithm^55^, improved walrus optimization algorithm^56^, chaotic swarm intelligence technique^57^, rime-ice algorithm^58^, exponential distribution optimizer^59^, chaotic Newton–Raphson-based optimizer^60^, modified manta ray foraging optimization^61^, chaotic Rao optimization algorithm^62^, improved light spectrum algorithm^63^, educational competition optimizer^64^, enhanced hunger games search algorithm^65^, hybrid slime mold enhanced convergent particle swarm optimizer^66^, differential evolution with dynamic crossover strategy^67^, parrot optimizer^68^, phototropic growth algorithm (PGA)^69^, attack defense strategy assisted osprey optimization algorithm (ADSOOA)^70^. A brief description of the published approaches employed to simulate the PEMFC is illustrated in Table 1.

**Table 1:** A brief description of the published approaches employed to simulate the PEMFC.

Ref	Years	Algorithm/improvement strategy	Target function	Dynamic validation	Remark
^37^	2021	Modified HHO/Fractional order	SSE	No	High precision of determination and Insufficient dynamic analysis
^42^	2022	Enhanced TSA/levy and the Weibull distribution functions	SSE	No	Greater convergence compared to TSA and concentrated on steady-state
^43^	2022	Enhanced Bald Eagle Algorithm/Levy function	SSE	No	Enhanced parameter evaluation and Absence of conditional assessment
^45^	2023	Improved Gorilla Troops/a Tangent Flight Strategy	SSE	No	Greater convergence and Restricted dynamic evaluation
^55^	2024	Enhanced Salp Swarm/opposition-based learning	SSE	No	A precise determination and Fixed applications in particular
^56^	2024	Improved Walrus Optimizer/Levy function	SSE	No	Enhanced quality of results and Study effect of different temperatures and pressure
^59^	2024	Exponential Distribution Optimizer/novel	SSE	Yes	Dependable performance under multiple conditions and Inability to use a dynamic model to examine temperature and pressure changes
^61^	2024	Modified Manta Ray Foraging/sin-cose approach	SSE	No	Improved reliability of extraction and Emphasis on steady states
^62^		Chaotic Rao Optimization/Chaotic maps	SSE	Yes	Both steady-state and dynamic validation, and More intricacy in the model
^65^	2025	Enhanced Hunger Games Search/hybrid with marine predator	SSE	No	Enhanced convergence speed, Restricted operating situation and high complexity
^67^	2025	Differential evolution/dynamic-Crossover	RMSE	No	Effective global search and Absence of dynamic PEMFC verification
^70^	2024	Improved Osprey Optimization/Attack-defense strategy	MSE	No	Increased precision of optimization and Restricted dynamic analysis
Proposed		SFOA/novel	SSE	Yes	Enhanced dynamic response, precision, and convergence

Depending on the above-mentioned research works, it is obvious that the majority of the reported metaheuristic optimization techniques have some limitations and restrictions when addressing the estimation of PEMFCs’ parameters as any variation in their values influences their technical indicators and exposes them to falling into best values leading to a minimum convergence rate. To address these limitations, the suggested SFOA is applied to improve the search process by adjusting population diversity and supporting the transition between global and local search stages. This allows the algorithm to better avoid early convergence while sustaining a competitive convergence speed. Accordingly, SFOA gives a more consistent and robust structure for precise PEMFC constraint estimation in comparison to prementioned approaches.

### Research contributions

The contributions of the present work are listed as follows:

- A novel implementation of the SFOA is proposed for accurate parameter extraction of PEMFC models, extending its application to fuel cell modelling.
- A comprehensive validation is conducted on multiple PEMFC stacks with different power ratings, demonstrating the robustness and generalization capability of the proposed approach.
- The proposed method is extensively compared with several recent and well-established optimization algorithms under identical conditions to ensure a fair and reliable performance assessment.
- The dynamic behavior of the PEMFC models is evaluated under varying operating conditions to verify the practical effectiveness of the proposed method.
- The statistical significance of the obtained results is validated using non-parametric tests, including Kruskal–Wallis and Friedman ANOVA.

### Research structure

The manuscript’s remainder is prepared as follows: Section "Mathematical Model of PEMFC" presents the PEMFC’s mathematical model. The explanation of optimization problem is exhibited in Section "Problem description". The suggested methodology combined with SFOA is presented in section "SFOA methodology". Section "Results and discussion" depicts the numerical and simulation outcomes and their discussions, while the research conclusions are clearly described in section "Conclusion".

## Mathematical Model of PEMFC

**Fig. 2:**
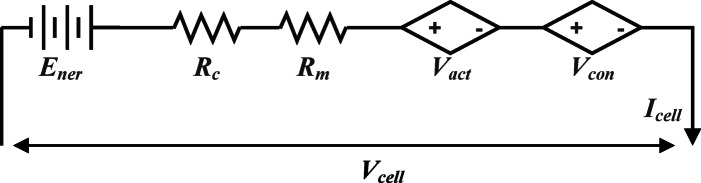
PEMFC electric circuit.

The comparable electrical circuit of the PEMFC stack is depicted in Fig. 2; it indicates the essential components that replicate the FC’s electrochemical reaction.

Due to ohmic, concentration, and activation losses, the open circuit voltage is less than the cell convertible voltage. As a result, the FC terminal voltage (${V}_{cell}$) and stack terminal voltage (${V}_{stack}$) can be evaluated as follows:

1 $${V}_{cell}={E}_{ner}-{V}_{ohm}-{V}_{con}-{V}_{act}$$

2 $${{V}_{stack}=V}_{cell}\times {N}_{c}$$

where ${E}_{ner}$ refers to the open circuit voltage, ${V}_{ohm}, {V}_{con}$, and ${V}_{act}$ indicate the ohmic, concentration, and activation voltage falls, respectively. The term ${N}_{c}$ symbolizes the cells number. The evaluate of ${E}_{ner}$ can be expressed as follows^71^:

3 $${E}_{ner}=1.229-8.5\times {10}^{-4}\left({T}_{c}-298.15\right)+4.385\times {10}^{-5}{T}_{c}\mathrm{ln}({P}_{a}\sqrt{{P}_{c}} )$$

where ${T}_{c}$ is the temperature of cell (K), ${P}_{a}$ and ${P}_{c}$ symbolize the pressures applied on the cathode and anode by oxygen and hydrogen gases, respectively.

**Fig. 3:**
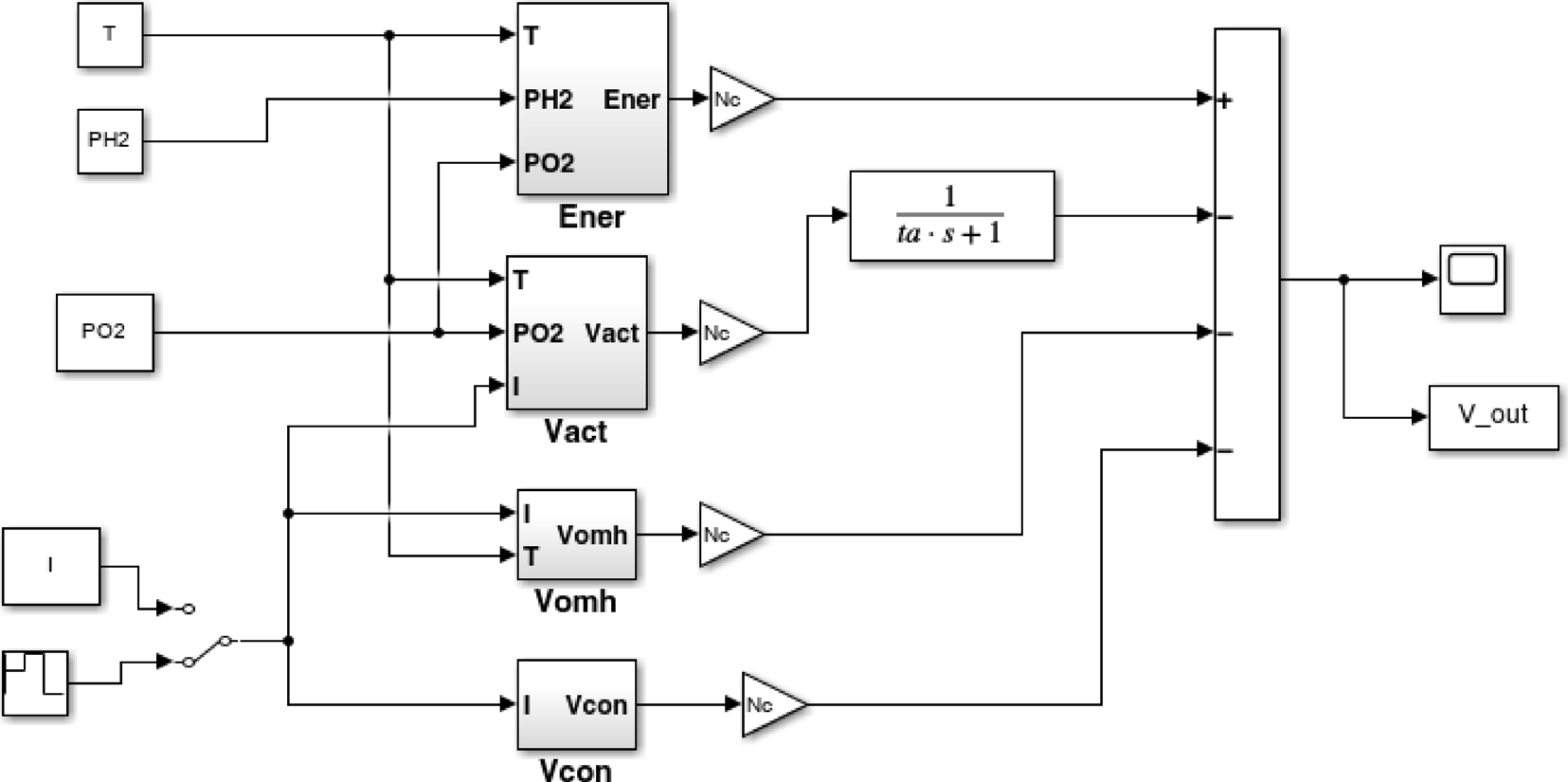
PEM fuel cell dynamic model.

The estimates of ${V}_{ohm}$ and ${V}_{con}$ can be written as^68^,

4 $${V}_{ohm}={I}_{c}\times \left(\frac{{\rho}_{m}\times l}{{A}_{c}}+{R}_{c}\right)$$

5 $${\rho}_{m}=\frac{181.6\times \left[1+0.03\times \left(\frac{{I}_{c}}{{A}_{c}}\right)+0.062\times \left(\frac{{T}_{c}}{303}\right)\times {\left(\frac{{I}_{c}}{{A}_{c}}\right)}^{2.5}\right]}{\left[\lambda -0.634-3\times \left(\frac{{I}_{c}}{{A}_{c}}\right)\right]\times exp\left[4.18\times \frac{{T}_{c}-303}{{T}_{c}}\right]}$$

6 $${V}_{con}=-\beta \times \mathrm{ln}\left(1-\frac{{I}_{c}}{{{A}_{c}\times J}_{max}}\right)$$

where ${I}_{c}$ indicates the FC current, ${A}_{c}$ means cell area (cm^2^), ${\rho}_{m}$ denotes the membrane resistivity, $l$ and ${R}_{c}$ symbolize the width of membrane (µm) and constant resistance, ${J}_{max}$ means the maximum density of current, and $\lambda$ is adjustable coefficients. The ${v}_{act}$ can be calculated as^72^,

7 $${v}_{act}=-\left[{\zeta}_{1}+{\zeta}_{2}\times {T}_{c}+{\zeta}_{3}\times {T}_{c}\times \mathrm{ln}\left({C}_{O2}\right)+{\zeta}_{4}\times {T}_{c}\times \mathrm{ln}\left({I}_{c}\right)\right]$$

8 $${C}_{O2}=\frac{{P}_{a}}{5.08\times {10}^{6}\times exp\left(\frac{-498}{{T}_{c}}\right)}$$

where ${\zeta}_{1}$, ${\zeta}_{2}$, ${\zeta}_{3}$, and ${\zeta}_{4}$ are factors and ${C}_{O2}$ signifies the oxygen intensity. The dynamic model of the PEMFC presented in Fig. 3 is used to evaluate the constructed model under various usage conditions.

## Problem description

The evaluation criteria are crucial for precisely describing the ungiven parameters through matching the calculated current–voltage (I-V) curve and the actual one. The PEMFC model’s ungiven parameters are ${\zeta}_{1}$*, *${\zeta}_{2}$*, *${\zeta}_{3}$*, *${\zeta}_{4}$*, *$\lambda$*, *${R}_{c}$*,* and $\beta$. The SSE between the calculated voltage (${V}_{cal}$) and the measured voltage (${V}_{mea}$) is the fitness function ($FF$) under consideration. An appropriate objective function simplifies parameter calculation and establishes between other model assessment approaches in terms of the limits of permissible outcomes, both quantitatively and qualitatively. The SSE is minimized at the number of collected values (${N}_{v}$) fed to the suggested SFOA; it can be described as follows:

9 $$FF=SSE(x)=min\sum_{i=1}^{{N}_{v}}{\left({V}_{mea}\left(i\right)-{V}_{cal}(i)\right)}^{2}$$

where $x$ represents the vector of parameters to be evaluated. Table 2 provides the parameter boundaries^61^.

**Table 2:** Boundaries of design parameters.

	${\zeta}_{1}$	${\zeta}_{2}$	${\zeta}_{3}$	${\zeta}_{4}$	$\lambda$	${R}_{c}$	$\beta$
*lb*	− 1.1997	8.00E−04	3.60E−05	− 2.60E−04	13	1.00E−04	0.0136
*ub*	− 0.8532	6.00E−03	9.80E−05	− 9.54E−05	23	8.00E−04	0.5

## SFOA methodology

The starfish optimization algorithm (SFOA) is a recent metaheuristic optimizer that mimics the exploring, food, and regenerative activities of starfish to resolve challenges in optimization^73^. The algorithm targets to find equilibrium between the exploitation phase, which directs the hunt regarding the best answer, and the exploration phase, which agrees to an accurate examination of the solution range. The primary refresh technique in the exploitation stage of SFOA is the hunting method, which utilizes a parallel both-directional hunting technique depending on the knowledge from both starfish to boost potential applicants to go in the path of superior locations. The locations of the starfish, which advocate various possibilities, are created at random within the specified search area during the initialization stage. This stage can be described as follows:

10 $${X}_{ij}={min}_{j}+r\left({max}_{j}-{min}_{j}\right)\quad i=\mathrm{1,2},\dots ,N j=\mathrm{1,2},\dots ,D$$

The minimum and maximum boundaries of the exploratory region are symbolized by ${min}_{j}$ and ${max}_{j}$, respectively. While ${X}_{ij}$ is the location of the *i*th starfish in the *j*th dimension, *r* refers to a random number in [0,1], $D$ and $N$ denotes the size of the problem dimension and population, respectively. The SFOA exploration stage mimics starfish’s five limbs’ ability to hunt for food. The five starfish limbs are utilized for assessing the limit of dimension. The exploratory region for problems with high dimensions, $D$ > 5, demanding that starfish utilize all five limbs in order to discover the region around them. Moreover, in order to direct the motion of their starfish limbs, they demand to identify which search agent location is optimal. In this stage, the location update calculation is as follows:

11 $$\left\{\begin{array}{l}{Y}_{i,p}^{T}={X}_{i,p}^{T}+\left(2r-1\right)\pi .\left({X}_{best ,p}^{T}-{X}_{i,p}^{T}\right)cos\left(\frac{\pi }{2}\cdot \frac{T}{{T}_{max}}\right),\hspace{0.25em}\hspace{0.25em}\hspace{0.25em}\hspace{0.25em}r\le 0.5\\ {Y}_{i,p}^{T}={X}_{i,p}^{T}-\left(2r-1\right)\pi .\left({X}_{best ,p}^{T}-{X}_{i,p}^{T}\right)sin\left(\frac{\pi }{2}\cdot \frac{T}{{T}_{max}}\right),\hspace{0.25em}\hspace{0.25em}\hspace{0.25em}\hspace{0.25em}r>0.5\end{array}\right.$$

where the present and attained locations of a starfish are symbolized by ${X}_{i,p}^{T}$ and ${Y}_{i,p}^{T},$ respectively. The present optimal location *p*-dimension is indicated as ${X}_{best ,p}^{T}$, where *p* is one of five chosen at random parameters from $D$ dimension. The terms $T$ and ${T}_{max}$ are the present and maximum iteration, respectively. On the additional hand, a one-dimensional discovery pattern is utilized for issues when D ≤ 5. In this situation, a starfish employs tracking data obtained from another starfish to move just one limb in attempt to find the food location. The location update is evaluated as follows:

12 $${Y}_{i,q}^{T}={E}_{t}{X}_{i,p}^{T}+{A}_{1}\left({X}_{{k}_{1},p}^{T}-{X}_{i,p}^{T}\right)+{A}_{2}\left({X}_{{k}_{2},p}^{T}-{X}_{i,p}^{T}\right)$$

13 $${E}_{t}=\frac{{T}_{max}-T}{{T}_{max}}\mathrm{c}\mathrm{o}\mathrm{s}\left(\frac{\pi }{2}\cdot \frac{T}{{T}_{max}}\right)$$

where the *p*-dimensional coordinates of two randomly chosen starfish are defined by ${X}_{{k}_{1},p}^{T}$ and ${X}_{{k}_{2},p}^{T}$, respectively while the term ${E}_{t}$ is the starfish power, ${A}_{1}$ and ${A}_{2}$ refer to the random number in [-1,1]. To enhance the search technique, SFOA involves mechanisms depicted from starfish prey and recuperation habits during the exploitation stage. Considering the prey attitude utilizes two-stage search technique, the starfish places are adjusted based on how far they are to the most well-known answer. The modified direct of every starfish’s can be computed as,

14 $${Y}_{i}^{T}={X}_{i}^{T}+{r}_{1}{d}_{m1}+{r}_{2}{d}_{m2}$$

15 $${d}_{m}=\left({X}_{\mathrm{b}\mathrm{e}\mathrm{s}\mathrm{t}}^{T}-{X}_{{m}_{p}}^{T}\right),m=1,\dots ,5$$

where ${r}_{1}$ and ${r}_{2}$ refers to the random factors in [0,1], while ${d}_{m1}$ and ${d}_{m2}$ denote the random chosen in ${d}_{m}$ which represents the five calculated separations between the ideal starfish in the group and different starfish. Because they move sluggishly, starfish are susceptible to other attackers when hunting. A starfish may lose one of its limbs while escaping from attack by predators. Hence, the final starfish in the population (*i* = *N*) experiences the recuperation stage of SFOA, this stage takes a long time. Therefore, during the recuperation stage the location is modified as,

16 $${Y}_{i}^{T}=\mathrm{e}\mathrm{x}\mathrm{p}\left(-T\times N/{T}_{max}\right){X}_{i}^{T}$$

If the updated locations given in Eqs. (14) and (16) are outside the limits of design parameters, they are modified based on the following formula:

17 $${X}_{i}^{T+1}=\left\{\begin{array}{cc}{Y}_{i}^{T}& min\le {Y}_{i}^{T}\le max\\ min& {Y}_{i}^{T}<min\\ max& {Y}_{i}^{T}>max\end{array}\right.$$

**Fig. 4:**
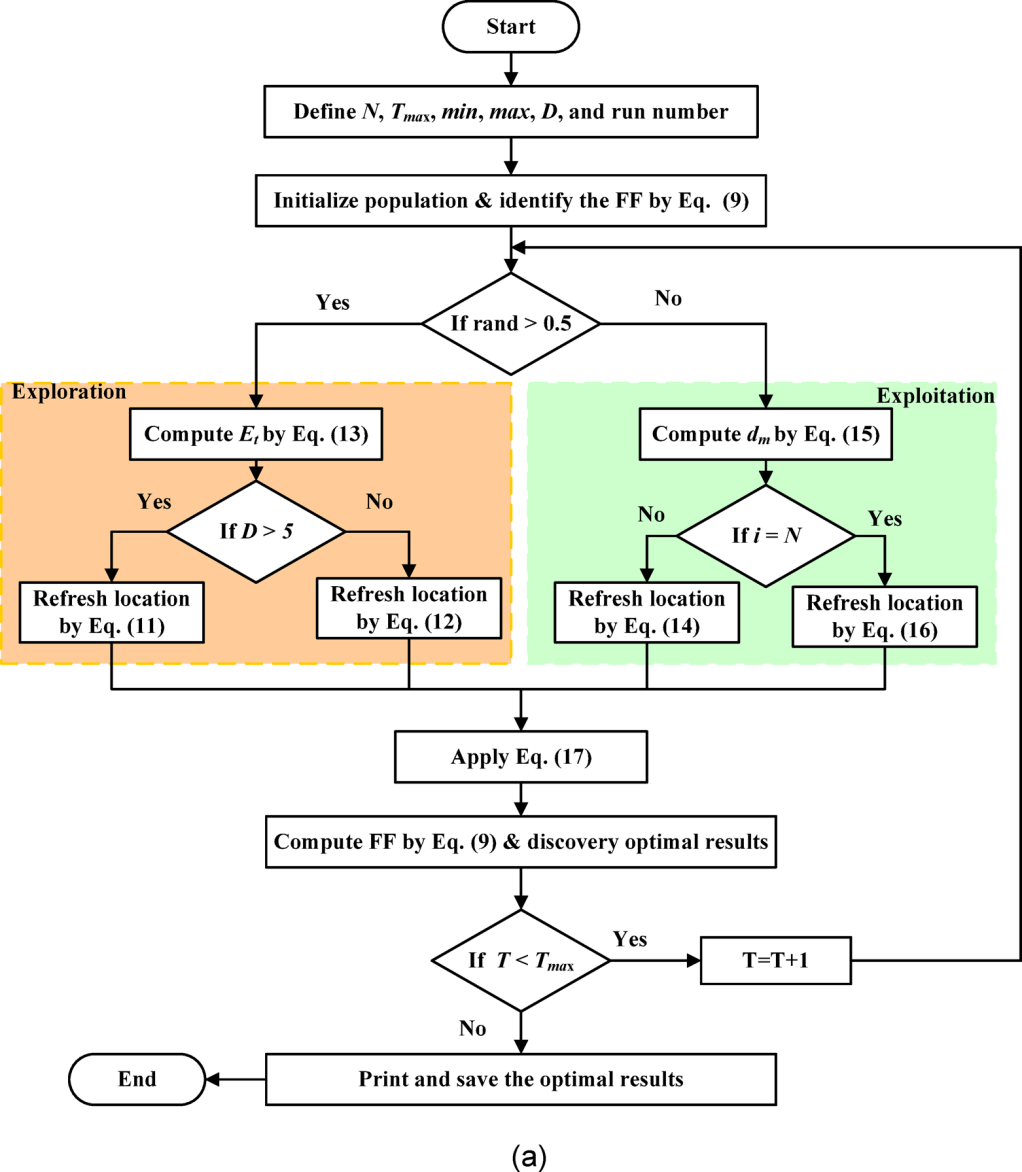

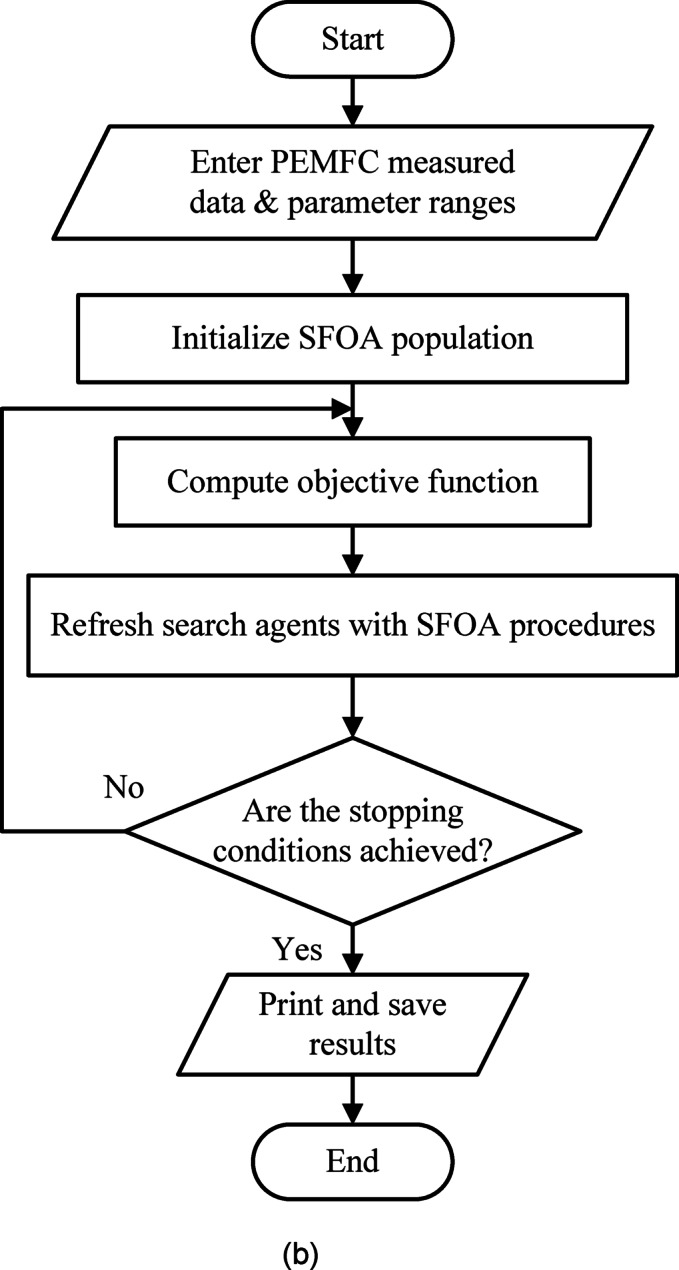
(**a**) SFOA flowchart and (**b**) flowchart of the suggested SFOA for PEMFC parameter estimation.

The program proceeds to run continuously to the completion criterion, usually the maximum number of iterations (${T}_{max}$). The SFOA flowchart with identifying optimal parameters for the PEMFC model is depicted in Fig. 4a.

The mechanism robustness and adaptability of the proposed SFOA for identifying the PEMFC parameters, whose model has high-dimensional nature, are highlighted as follows:

- The SFOA’s design depends on investigating the extremely non-linear characteristics and multi-variable complexity of the PEMFC that typically limit traditional optimizers by assessing a multi-dimensional exploration, which is finding the seven unknown parameters for the PEMFC as described in Table 1 through assigning a five-limb hunting strategy, which allows the algorithm to discover the search region more thoroughly.
- It investigates a parallel both-directional hunting technique during the exploitation phase. By employing knowledge from multiple search agents at the same time, it efficiently enhances potential candidates toward superior locations with fewer iterations, which is important for navigating the complex, non-linear equations of the PEMFC.
- It has a recuperation mechanism by modifying the location of the least-performing agent to guarantees the population preserves diversity and avoids local optima, a public failure spot for traditional optimization techniques in non-linear identification problems.

Figure 4b illustrates a flowchart of the suggested SFOA for PEMFC parameter estimation. The Pseudo code of the suggested solution methodology incorporated SFOA is given in Algorithm 1.

**Algorithm 1:**
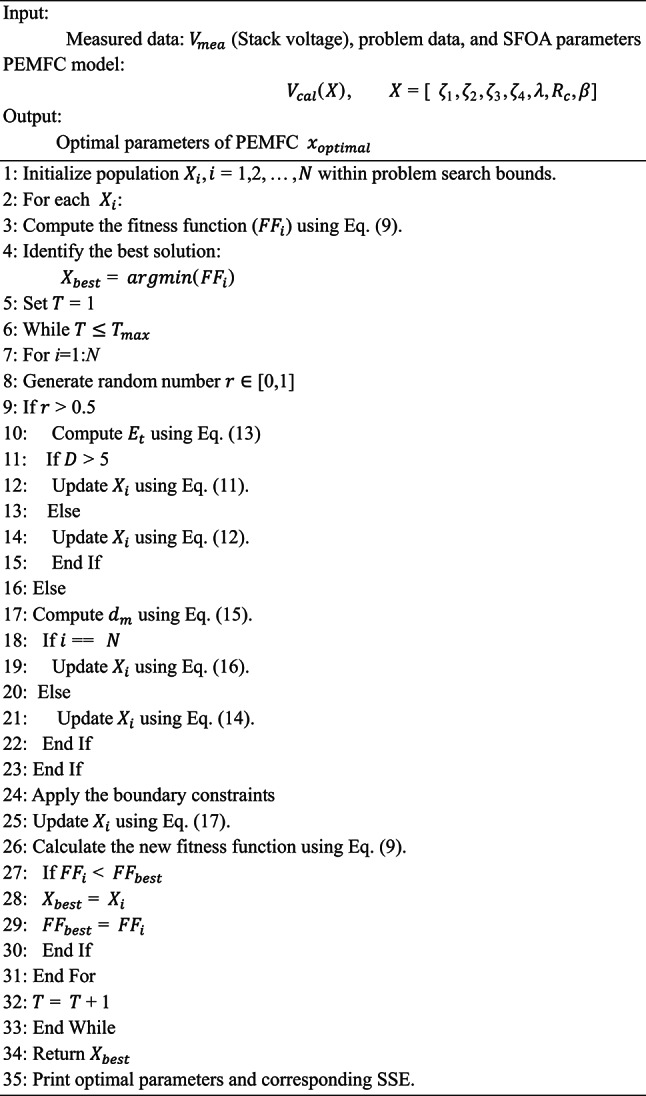
Pseudo code of the proposed solution methodology incorporated SFOA

## Results and discussion

In this section, the suggested SFOA is employed to assess the parameters of five distinct FCs: Horizon_H12, Horizon H-1000XP PEMFC stack, Temasek_1kw, SR-12PEM 500-W, and BCS-500W PEMFC stacks. The aim of selecting five distinct structures is to evaluate the proposed approach’s effectiveness on a variety of structures with different characteristics. Table 3 lists the technical specifications of the PEMFCs under consideration. Because it is evident that the working circumstances of the chosen PEMFCs vary, the audience may examine the feasibility of the approach being suggested. Furthermore, these FCs are chosen since the described techniques incorporate experimental data. Each cell’s functioning circumstances vary depending on the kind of material used to make the proton membrane, which influences the operational temperature, energy density, and pressure on both cathode and anode as well as the proton conductivity and structure of the cells. In general, the temperature in these types of cells shouldn’t go above 100 °C to prevent membrane dryness and maintain it wet^53^. The proposed SFOA outcomes are compared to informed methods of seagull optimization algorithm (SOA), escape optimizer (ESC), gold rush optimizer (GRO), tunicate swarm algorithm (TSA), beluga whale optimization (BWO), and success history intelligent optimizer (SHIO). These algorithms were precisely chosen as they represent diverse optimization strategies and search behaviors. To ensure fair evaluation, the approaches are constructed for 30 separate runs with identical population size and iteration count of 30 and 1000, respectively. This various selection guarantees that the comparison covers diverse algorithmic characteristics, including robustness, convergence speed, and the capability to avoid premature convergence, by this means offering a consistent benchmark to estimate the effectiveness of the proposed SFOA in solving the PEMFC parameter identification problem. All algorithms’ parameter choices are chosen according to their frequently used values in the literature to ensure a fair comparison, and original sources and the parameters of all employed optimizer are illustrated in Table 4.

**Table 3:** The arrangements of the studied PEMFCs.

Parameters	Horizon_H12	Horizon H-1000XP PEMFC stack	BCS-500w	Temasek_1kw	SR-12 500W
*N*_*cell*_	13	50	32	20	48
*A* (cm^2^)	8.1	77	64	150	62.5
*J*_*max*_ (A/cm^2^)	0.86	0.56	0.469	1.5	0.672
*l* (µm)	25	125	178	51	25
*T* (K)	302	328	333	323	343
*P*_*H2*_ (bar)	0.5	1	1.0	0.5	1.47628
*P*_*O2*_ (bar)	1	0.56	0.2095	0.5	0.2095
*R*_*Ha*_		1	1.0	1.0	1.0
*R*_*Hc*_		1	1.0	1.0	1.0

**Table 4:** The optimization procedures’ characteristics.

Algorithm	Parameters
SFOA	*N* = 30, *T*_*ma*x_ = 1000, *run no*. = 30, *G*_*p*_ = 0.5
SOA	*N* = 30, *T*_*ma*x_ = 1000, *run no*. = 30, *b* = 1, *r*_*1*_ = *r*_*2*_ = rand(0,1), *c*_*1*_ = 2*r2
TSA	*N* = 30, *T*_*ma*x_ = 1000, *run no*. = 30, *x*_*min*_ = 1, *x*_*ma*x_ = 4, *c*_*2*_ = *c*_*3*_ = rand(0,1)
GRO	*N* = 30, *T*_*ma*x_ = 1000, *run no*. = 30, *r*_*1*_ = *r*_*2*_ = rand(0,1), *c*_*1*_ = 2*r_*2*_*, δi* = *2, δf* = *1/T*_*max*_
ESC	*N* = 30, *T*_*ma*x_ = 1000, *run no*. = 30, *a* = 0.15, *b* = 0.35, *β* = 1.5, *M*_*p*_ = 0.5
BWO	*N* = 30, *T*_*ma*x_ = 1000, *run no*. = 30, *w*_*f*_ = [0.1 0.05], *r*_*1*_ = *r*_*2*_ = *r*_*3*_ = *r*_*4*_ = rand(0,1),
SHIO	*N* = 30, *T*_*ma*x_ = 1000, *run no*. = 30, *α* = 1.5

### The horizon H-12 PEMFC

**Fig. 5:**
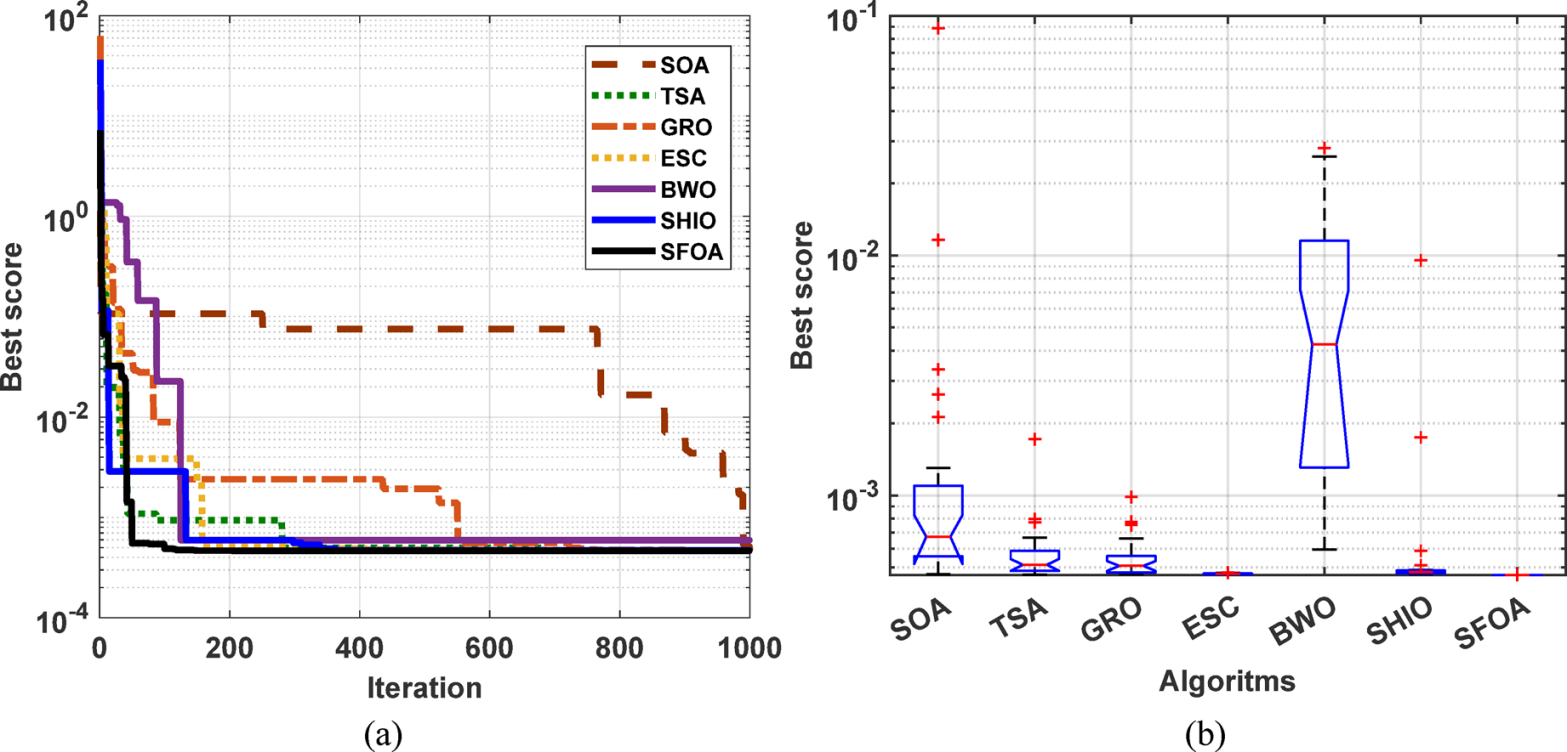
Variations of (**a**) convergence curves, (**b**) boxplots of algorithms applied on Horizon H-12 PEMFC.

The first considered fuel cell is the Horizon H-12 PEMFC. In this instance, 13 fuel cells with series connections are demonstrated. The H-12 PEMFC has a thickness of 25 μm and a surface area of 8.1 cm^2^. The highest possible current density has been fixed at 0.86 A/cm^2^. The development of convergence of the suggested SFOA algorithm over 1000 iterations is depicted in Fig. 5a. Also Fig. 5b shows boxplots of the pervious optimization techniques. Although the findings demonstrate that the assessed optimization techniques performed similarly in the present instance, the SFOA approach required fewer iterations to reach convergence. Additionally, the SFOA algorithm boxplot is the narrow in comparison to the others, indicating that it is the best through the executed runs. In addition, Table 5 provides additional information and comparisons of the SFOA to other existing optimization techniques during searching of the seven parameters of the considered Horizon H-12 PEMFC. The SFOA required only about 7% of the number of iterations to go lower than the outcomes attained by the other approaches. The suggested SFOA attained the greatest fitness value of 4.662696E-04, outperforming all the remaining methods. However, the poorest score is 9.68500E-02, acquired with ETSO^42^. Furthermore, the approaches investigated are evaluated by computing several statistical parameters like best, worst, average (Ave), and standard deviation (Std), shown in Table 6. It is obvious that the intended SFOA yielded the best outcomes among the others with a Std of 1.797814E-16, whereas SOA has the poorest Std of 1.609958E-02. The findings reveal that, the suggested SFOA performed effectively in predicting the parameters of the Horizon H-12 PEMFC.

**Table 5:** The optimum parameters of Horizon H-12 PEMFC based on the proposed SFOA and other algorithms.

Algorithm	*ζ*_*1*_	*ζ*_*2*_	*ζ*_*3*_	*ζ*_*4*_	*λ*	*R*_*c*_	*b*	*SSE*
SFOA	− 9.2627E−01	2.3861E−03	7.4227E−05	− 9.5400E−05	13.000	8.0000E−04	1.6548E−01	**4.662696E**−**04**
SOA	− 1.1997E+0	3.3442E−03	7.7914E−05	− 9.5400E−05	14.466	7.5113E−04	1.6885E−01	4.686110E−04
TSA	− 1.0106E+0	2.2371E−03	4.4606E−05	− 9.5400E−05	13.000	7.0514E−04	1.6621E−01	4.676408E−04
GRO	− 1.0018E+0	2.3415E−03	5.3845E−05	− 9.5416E−05	14.266	5.0942E−04	1.7007E−01	4.688912E−04
ESC	− 1.1005E+0	2.8276E−03	6.4883E−05	− 9.5400E−05	15.149	6.8307E−04	1.7068E−01	4.684483E−04
BWO	− 8.5320E−01	1.5923E−03	3.6000E−05	− 9.5400E−05	13.000	1.8643E−04	1.6922E−01	5.941155E−04
SHIO	− 8.6963E−01	1.7230E−03	4.1325E−05	− 9.5460E−05	14.969	5.7994E−04	1.7100E−01	4.694446E−04
ETSO^42^	− 1.0323E+0	2.7297E−03	7.7200E−05	− 9.5400E−05	22.9990	1.1242E−04	1.8689E−01	9.65300E−02
TSO^42^	− 8.5320E−01	1.5719E−03	3.6100E−05	− 9.5400E−05	13.0244	3.2787E−04	1.7527E−01	9.68500E−02
EBES^43^	− 1.1279E+0	2.7945E+03	5.6100E−05	− 6.7800E−05	51.5950	9.6800E−04	2.6133E−01	7.486410E−02
WOA^74^	− 1.1870E+0	2.6697E+03	3.6000E−05	− 9.5400E−05	13.8240	8.0000E−04	1.5980E−01	1.160000E−01

Significant values are in [bold].

**Table 6:** Worst, Ave, best, and Std of the considered optimization algorithm of the Horizon H-12 PEMFC.

Algorithm	Worst	Ave	Best	Std
SFOA	**4.662696E−04**	**4.662696E−04**	**4.662696E−04**	**1.797814E−16**
SOA	8.874861E−02	4.205735E−03	4.686110E−04	1.609958E−02
TSA	1.716607E−03	5.815053E−04	4.676408E−04	2.300936E−04
GRO	9.847084E−04	5.582565E−04	4.688912E−04	1.272896E−04
ESC	4.761196E−04	4.713107E−04	4.684483E−04	1.877796E−06
BWO	2.804121E−02	7.429789E−03	5.941155E−04	8.133797E−03
SHIO	9.533227E−03	8.276918E−04	4.694446E−04	1.660446E−03

Significant values are in [bold].

**Fig. 6:**
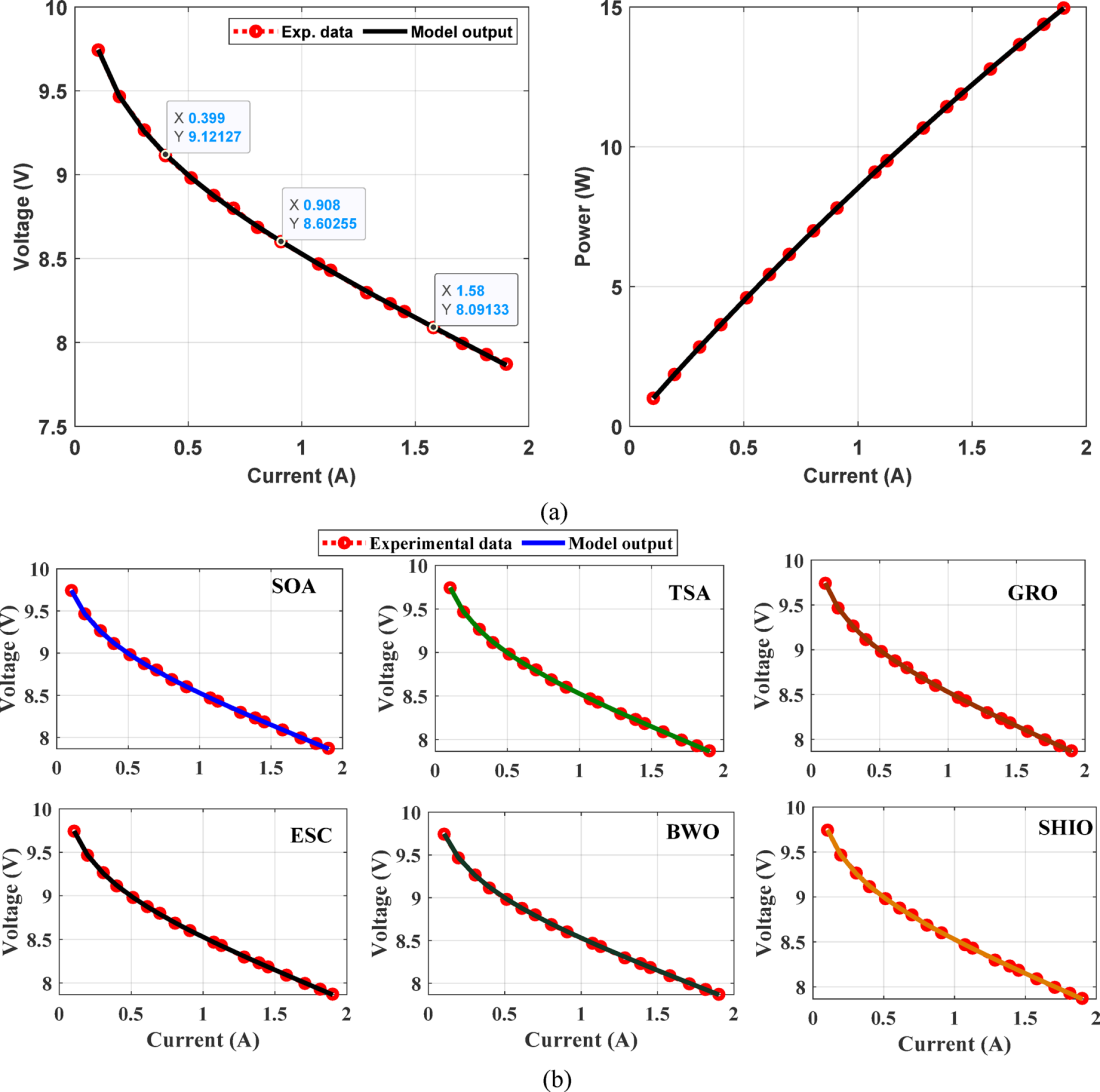
(**a**) I–V and I–P polarization curves of the Horizon H-12 PEMFC. (**b**) I–V polarization curves of the Horizon H-12 PEMFC for other optimizers.

Figure 6a displays the observed and evaluated I-V and current-power (I-P) polarization graphs produced using the suggested SFOA for the Horizon H-12 PEMFC. It should be highlighted that, the strong convergence between the two data points shows that the proposed technique is effective in calculating the correct parameters of the Horizon H-12 PEMFC equivalent circuit. The measured and assessed I-V polarization graphs created with the various optimizers for the Horizon H-12 PEMFC are demonstrated in Fig. 6b.

**Fig. 7:**
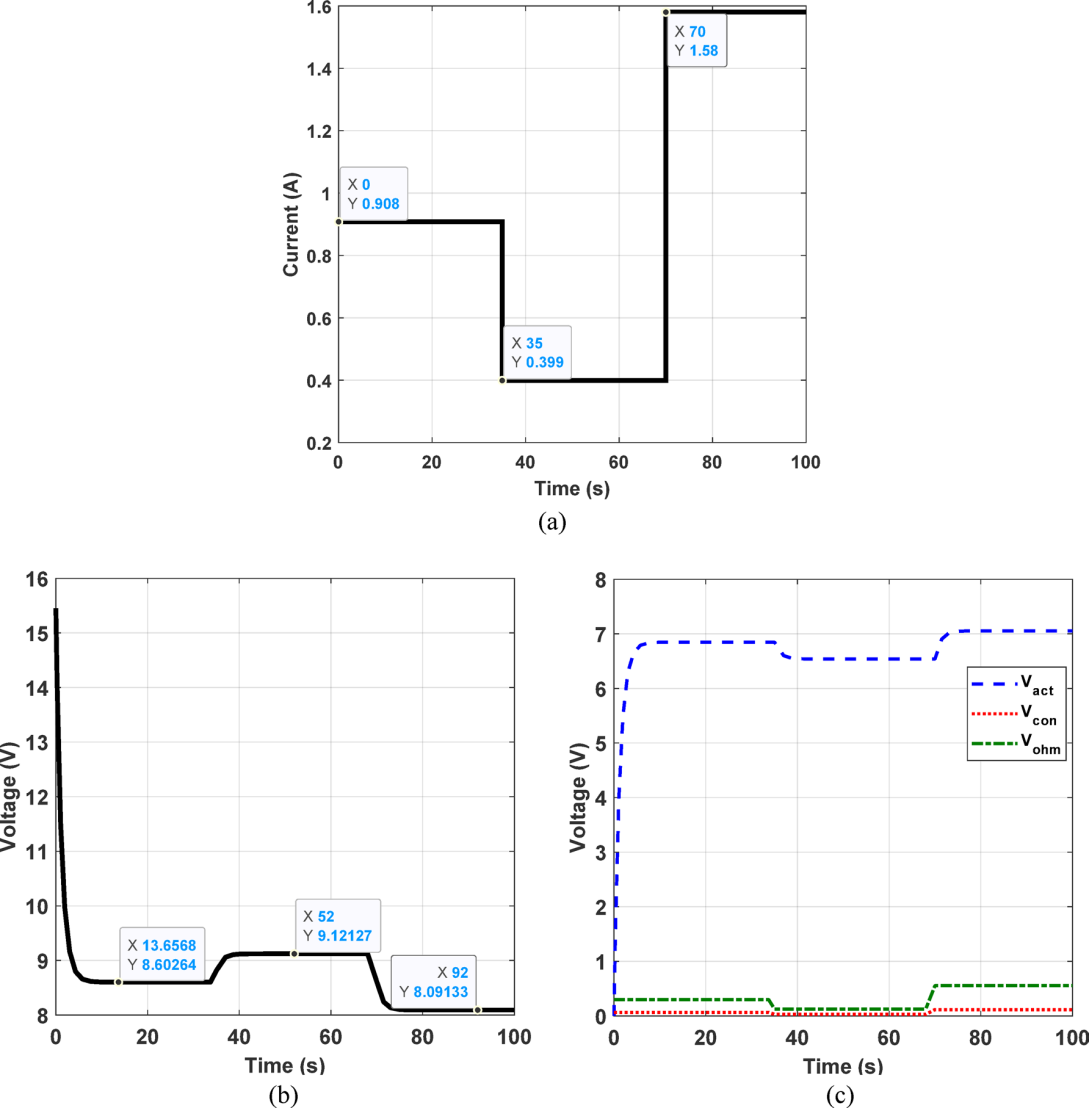
The Horizon H-12 PEMFC (**a**) current, (**b**) terminal voltage, and (**c**) time responses of activation, concentration, and ohmic.

However, the load perturbation delivered to the Horizon H-12 PEMFC dynamic simulation at 333 K with an anode pressure of 1 bar and cathode pressure of 0.2095 bar is depicted in Fig. 7a. With sampling time of 35 s, the load current’s peak values are 0.908 A, 0.399 A, and 1.58 A. Figure 7b displays the terminal’s voltage of the Horizon H-12 PEMFC. The model’s output voltages are 8.60264 V and 9.12127 V when the current is 0.908 A and 0.399 A. Ultimately, when the load current diminishes to 1.58 A, the terminal voltage falls to 8.0913 V. These findings in Fig. 7b are consistent with those in Fig. 7a. The terminal voltage value is impacted by the model’s response to any variation in the load current. Figure 7c shows the activation, concentration, and ohmic voltage time responses at the load disturbance under consideration.

### Horizon H-1000XP PEMFC stack

**Fig. 8:**
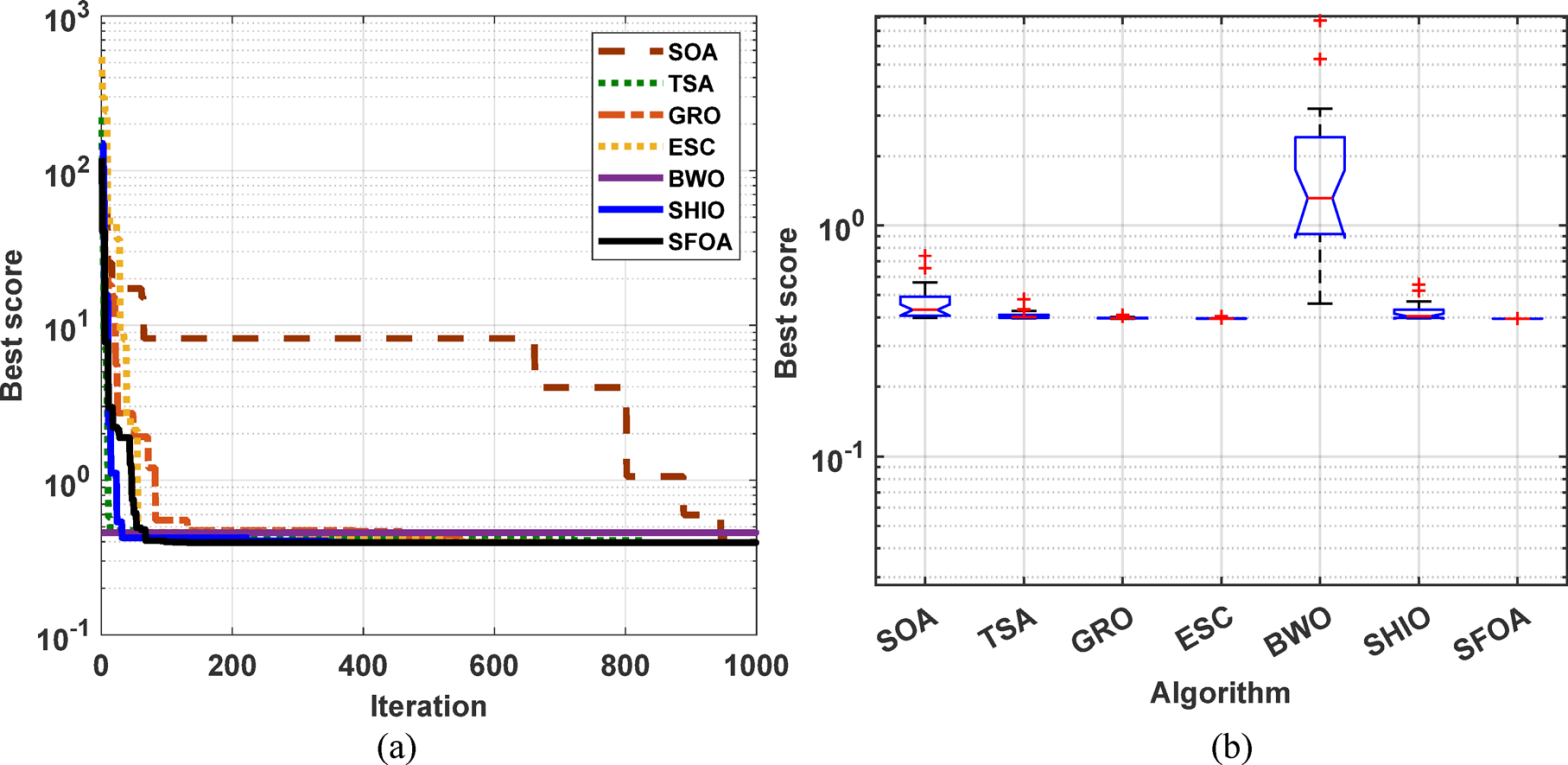
Variations of (**a**) best score versus iteration number, (**b**) boxplots for approaches applied to Horizon H-1000XP PEMFC stack.

**Fig. 9:**
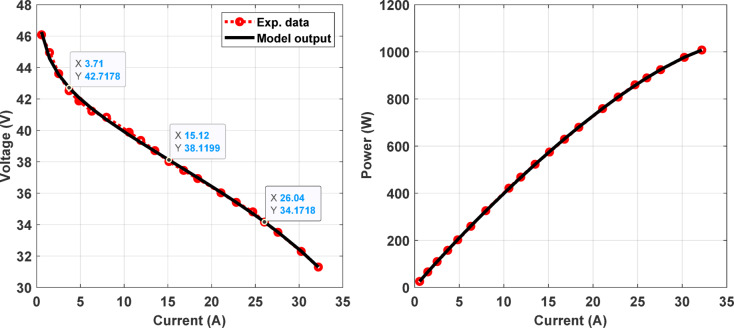
I–V and I–P polarization curves of Horizon H-1000XP PEMFC stack.

The second cell evaluated in this study is Horizon H-1000XP PEMFC stack. The suggested SFOA and other algorithms are applied to select the optimal parameters of the evaluated Horizon H-1000XP PEMFC stack. Furthermore, the optimum parameters and SSE values are presented in Table 7. The best records are 3.948527E-01, 3.948650E-01, and 3.949361E-01 resulted from applying the proposed SFOA , GRO, and ESC, respectively. While the highest record is 4.449000E-01 obtained through WOA^75^. Additionally, the statical analysis of the proposed SFOA algorithm and other approaches applied on Horizon H-1000XP PEMFC stack are recorded in Table 8. Figure 8a,b demonstrate the variations of highest score with iteration numbers as well as boxplots. According to Fig. 8a, the suggested SFOA approach achieved a superior fitness index in 70 iterations demonstrating that it is the most rapid way among the alternatives. Figure 9 depicts the observed and evaluated I-P and I-V curves acquired using the suggested SFOA. Each curve exhibits remarkable convergence.

**Table 7:** The optimized parameters for Horizon H-1000XP PEMFC stack based on the suggested SFOA and other algorithms.

Algorithm	*ζ*_*1*_	*ζ*_*2*_	*ζ*_*3*_	*ζ*_*4*_	*λ*	*R*_*c*_	*b*	*SSE*
SFOA	− 1.0541E + 0	2.8639E−03	3.6027E−05	− 9.5400E−05	24.000	4.8443E−04	9.2344E−02	**3.948527E−01**
SOA	− 9.5540E−01	2.7456E−03	4.8709E−05	− 9.5400E−05	20.305	2.4139E−04	9.3278E−02	3.973676E−01
TSA	− 1.1984E + 0	3.5928E−03	5.5907E−05	− 9.5400E−05	16.167	1.2844E−04	8.7840E−02	3.955922E−01
GRO	− 1.0853E + 0	3.7933E−03	9.3459E−05	− 9.5401E−05	22.730	4.4180E−04	9.1909E−02	3.948650E−01
ESC	− 1.0892E + 0	3.8691E−03	9.7863E−05	− 9.5414E−05	20.760	3.3095E−04	9.1902E−02	3.949361E−01
BWO	− 1.0307E + 0	2.9727E−03	4.8541E−05	− 9.7270E−05	17.871	3.1582E−04	8.4395E−02	4.588490E−01
SHIO	− 1.1276E + 0	3.2193E−03	4.5049E−05	− 9.5965E−05	22.337	3.8206E−04	9.2484E−02	3.952827E−01
IWOA^75^	− 1.0731E + 0	3.1000E−03	4.0000E−05	− 1.0000E−04	24.0000	1.0000E−04	9.8300E−02	4.449000E−01
WOA^75^	− 8.532E−01	2.5000E−03	1.0000E−04	− 1.0000E−04	23.9949	1.0000E−04	9.8200E−02	4.449000E−01
CNRBO3^60^	− 9.0175E−01	2.4490E−03	3.6970E−05	− 9.9688E−05	23.9999	1.3104E−04	8.9559E−02	4.36450E−01
RBO^60^	− 1.1997E−01	3.8412E−03	7.1452E−05	− 9.6037E−05	18.9631	2.3415E−04	9.7151E−02	4.37290E−01

Significant values are in [bold].

**Table 8:** Statistical indicators of different optimizers applied on Horizon H-1000XP PEMFC stack.

Algorithm	Worst	Ave	Best	Std
SFOA	**3.948540E−01**	**3.948527E−01**	**3.948527E−01**	**2.499297E−07**
SOA	7.389200E−01	4.622186E−01	3.973676E−01	7.988961E−02
TSA	4.797827E−01	4.077838E−01	3.955922E−01	1.727428E−02
GRO	4.092718E−01	3.980527E−01	3.948650E−01	3.739384E−03
ESC	4.041206E−01	3.957535E−01	3.949361E−01	1.840767E−03
BWO	7.736115E+00	1.897883E+00	4.588490E−01	1.535686E+00
SHIO	5.554652E−01	4.214596E−01	3.952827E−01	3.853862E−02

Significant values are in [bold].

**Fig. 10:**
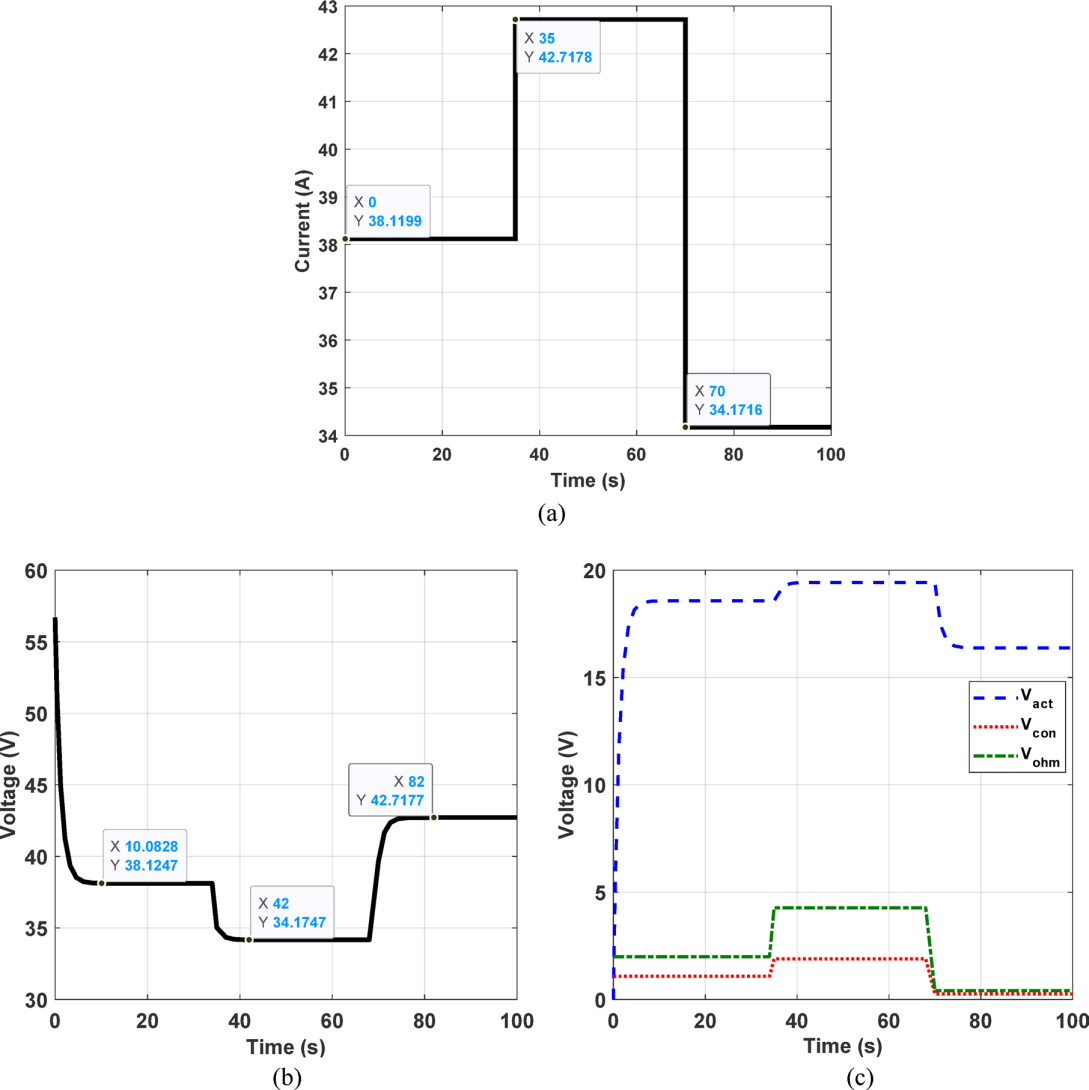
The Horizon H-1000XP PEMFC (**a**) current, (**b**) terminal voltage, and (**c**) time responses of activation, concentration, and ohmic.

However, Fig. 10a depicts the load perturbation applied to the Horizon H-1000XP PEMFC stack. With a sample interval of 35 s, the load current peaks at 15.12 A, 26.04 A, and 3.71 A. Figure 10b shows the terminal voltage of the Horizon H-1000XP PEMFC. When the current is 15.12 A and 26.04 A, the model produces output voltages of 38.1247 V and 34.1747 V, respectively. Finally, when the load current is reduced to 3.71 A, the terminal voltage reaches 42.7177 V. The outcomes shown in Fig. 10b are comparable with those in Fig. 10a. The model’s response to any fluctuation in load current has an influence on the terminal voltage value. Figure 10c depicts the activation, ohmic, and concentration voltage time responses to the load perturbation being considered.

### Temasek_1kw PEMFC

**Fig. 11:**
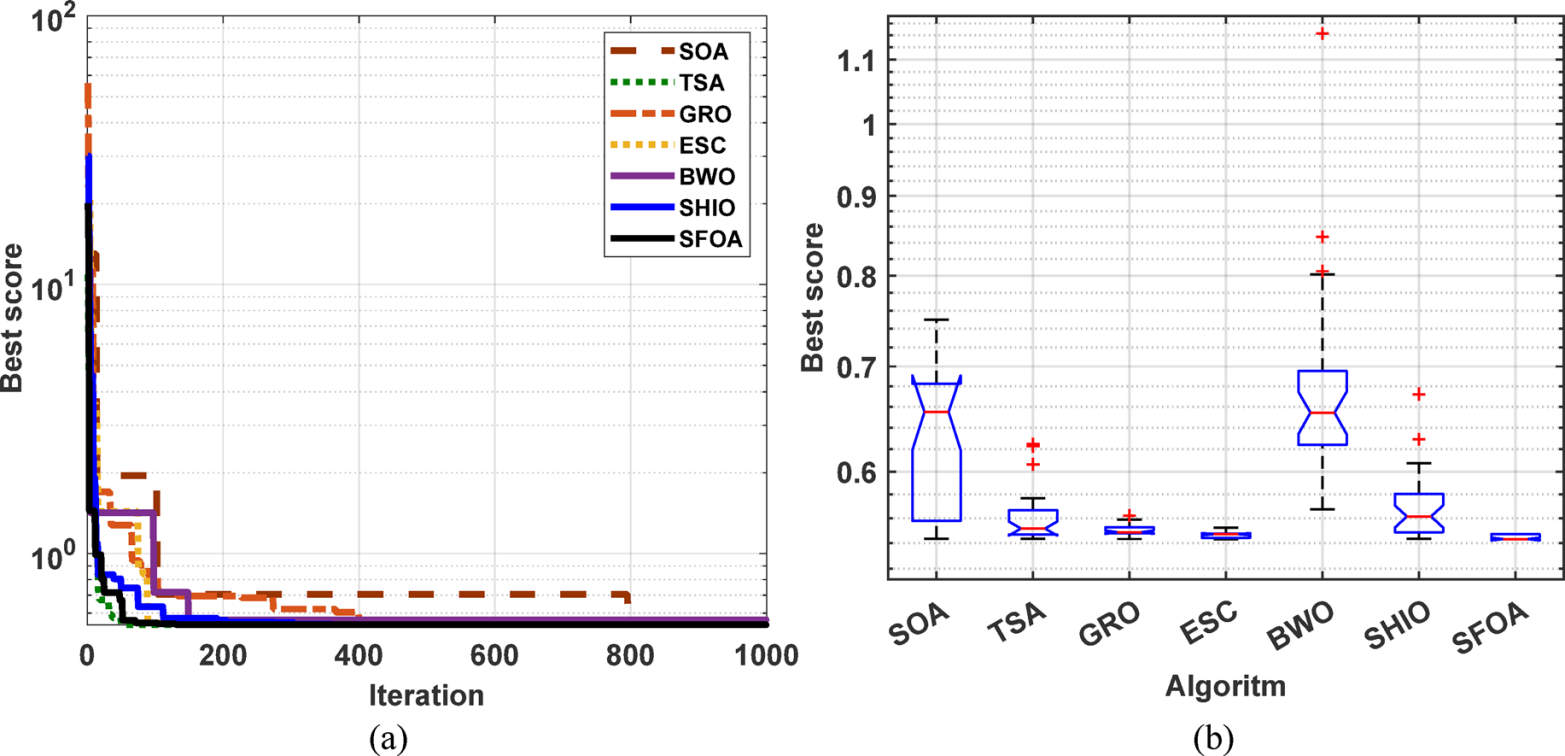
Variations of (**a**) best score versus iteration number, (**b**) boxplots for approaches applied on Temasek_1kw PEMFC.

**Fig. 12:**
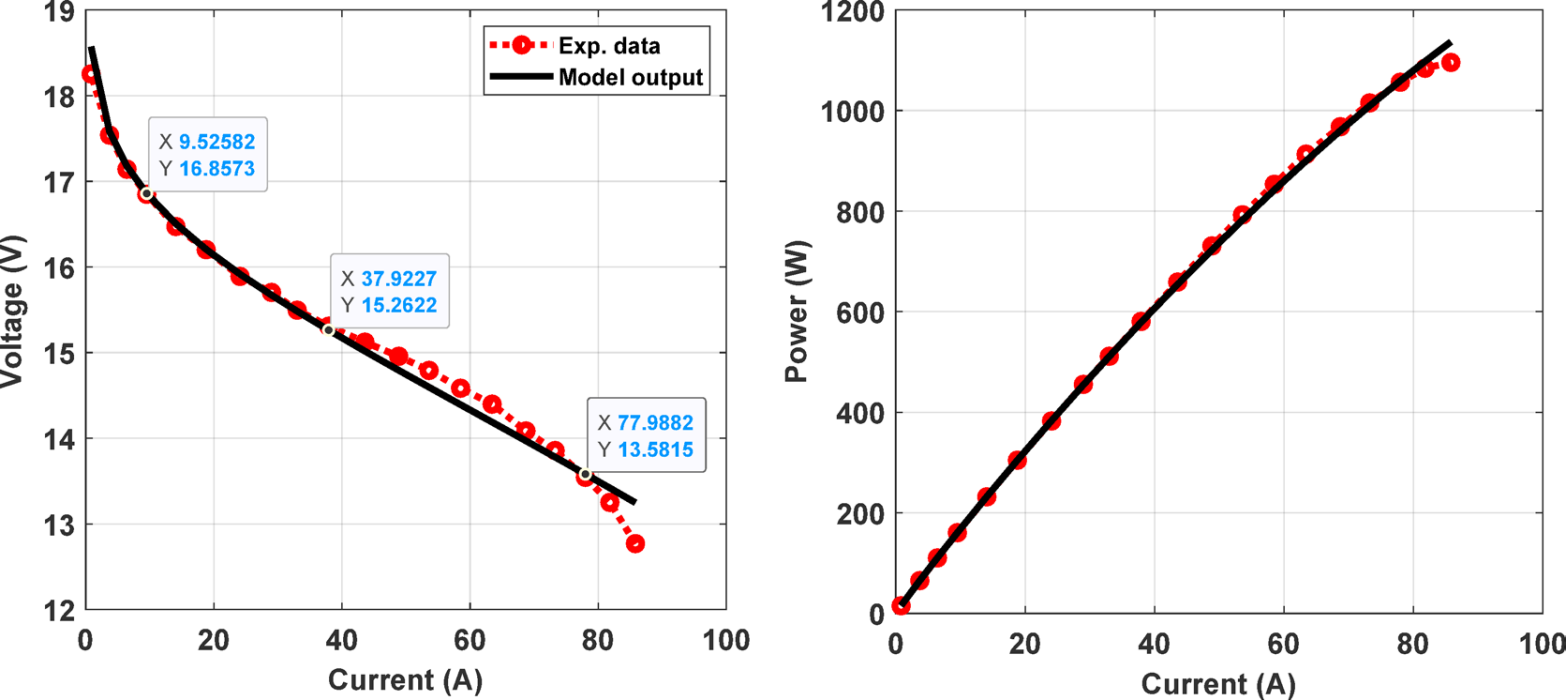
The I–V and I–P polarization curves of Temasek_1kw PEMFC.

**Fig. 13:**
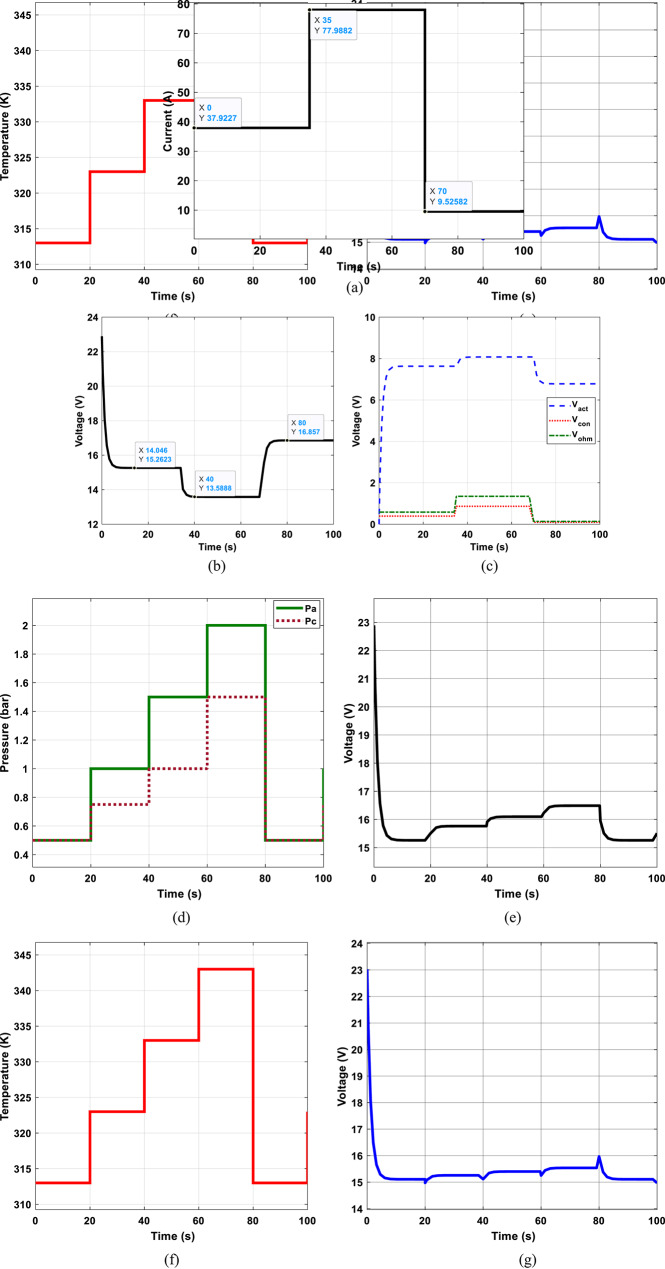
The Temasek_1kw PEMFC (**a**) current, (**b**) terminal voltage, (**c**) time responses of activation, concentration, and ohmic, (**d**) disturbance pressure, (e) terminal voltage under different pressure, (**f**) change of temperature, and (**g**) terminal voltage under different temperature.

In this section, the third FC stack used for confirming the identification technique based on the SFOA is the Temasek 1 kW PEMFC. The optimum parameters of the considered FC based on the SFOA algorithm and other approaches are listed in Table 9. Additionally, the statical results are shown in Table 10. It is noticeable that the suggested SFOA generated the best outcomes among the others with an Std of 1.878254E-03, whereas BWO has the weakest Std of 1.125103E-01. The conclusions reveal that the suggested SFOA performed effectively in expecting the parameters of Temasek 1 kW PEMFC. The SFOA addresses the best outcomes in comparison with other methods. The resulting convergence graphs and boxplot are shown in Fig. 11a, b. The boxplots confirm the stability of the SFOA method. It required just roughly 6% of the iterations to get better results than the other techniques. The recommended approach achieved the highest fitness value of 5.4322160E-01 beating all other approaches. Nonetheless, the lowest fitness score of 7.986870E-01, is obtained with Rao-3^62^. The evaluated I-P and I-V graphs produced using the SFOA are depicted in Fig. 12. It should be emphasized that the convergence between the two data points is identical. This shows that the suggested algorithm is effective in assessing the correct parameters of the Temasek_1kw PEMFC equivalent circuit. Additionally, Fig. 13a,b show the load perturbation as well as the terminal voltages of the Temasek_1kw PEMFC. The load current peaks are 37.9227 A, 77.9882 A, and 9.5258 A, respectively, considering interval time occurrence every 35 s. The output voltage values with respect to the duration intervals related to the current are 15.2623 V, 13.5888 V, and 16.857 V, respectively. Additionally, Fig. 13c depicts the activation, ohmic, and concentration voltage time responses to the load perturbation being considered. The impacts of temperature and pressure changes were examined independently while maintaining a constant load current at 38 A. Figure 13d displays the pressure fluctuation profile, while Fig. 13e displays an increase in output voltage as pressure rises. In addition, Fig. 13f illustrates the temperature change over time, while Fig. 13g indicates that the output voltage grows with temperature. As illustrated in Fig. 13e,g, a rise in temperature improves membrane conductivity and reaction kinetics, leading to better dynamic activity, while an increase in pressure improves the voltage response because of larger reactant partial pressures.

**Table 9:** The optimized parameters Temasek_1kw PEMFC acquired based on the suggested SFOA and others.

Optimizer	*ζ*_*1*_	*ζ*_*2*_	*ζ*_*3*_	*ζ*_*4*_	*λ*	*R*_*c*_	*b*	*SSE*
SFOA	− 8.7368E−01	3.3509E−03	9.7330E−05	− 9.5400E−05	13.000	1.0000E−04	1.5820E−01	**5.4322160E−01**
SOA	− 1.1911E+00	3.4007E−03	3.6000E−05	− 9.5400E−05	13.000	1.0063E−04	1.5777E−01	5.434810E−01
TSA	− 8.5320E−01	3.0191E−03	7.9693E−05	− 9.5400E−05	13.000	1.0000E−04	1.5729E−01	5.435740E−01
GRO	− 9.2095E−01	3.0402E−03	6.7300E−05	− 9.5400E−05	13.000	1.0004E−04	1.5792E−01	5.432465E−01
ESC	− 1.0229E+00	3.8169E−03	9.7603E−05	− 9.5400E−05	13.000	1.0000E−04	1.5822E−01	5.432223E−01
BWO	− 8.5320E−01	2.8052E−03	6.5554E−05	− 9.5400E−05	13.000	1.5350E−04	1.4412E−01	5.675456E−01
SHIO	− 1.1997E+00	3.8304E−03	6.2460E−05	− 9.5400E−05	13.000	1.0037E−04	1.5832E−01	5.434784E−01
MMRFO^61^	− 9.8120E−01	3.7282E−03	6.4217E−05	− 2.3043E−04	13.0126	1.0002E−04	6.6683E−02	7.909900E−01
CNRBO3^60^	− 1.18591E+0	4.2241E−03	6.1662E−05	− 2.2765E−04	10.0000	1.0000E−04	3.9582E−02	7.627700E−01
Rao-3^62^	− 8.5320E−01	4.0192E−03	9.8000E−05	− 9.5400E−04	22.9970	1.1428E−04	5.4306E−02	7.986870E−01

Significant values are in [bold].

**Table 10:** Statistical indicators of different optimizers applied on Temasek_1kw PEMFC.

	Worst	Ave	Best	Std
SFOA	**5.471390E−01**	**5.445274E−01**	**5.432216E−01**	**1.878254E−03**
SOA	7.498509E−01	6.305749E−01	5.434810E−01	6.435012E−02
TSA	6.248693E−01	5.604752E−01	5.435740E−01	2.171785E−02
GRO	5.622584E−01	5.501910E−01	5.432465E−01	4.175140E−03
ESC	5.523187E−01	5.461920E−01	5.432223E−01	2.171335E−03
BWO	1.143023E+00	6.827290E−01	5.675456E−01	1.125103E−01
SHIO	6.722375E−01	5.698939E−01	5.434784E−01	2.831528E−02

Significant values are in [bold].

### The SR-12PEM 500-W

**Fig. 14:**
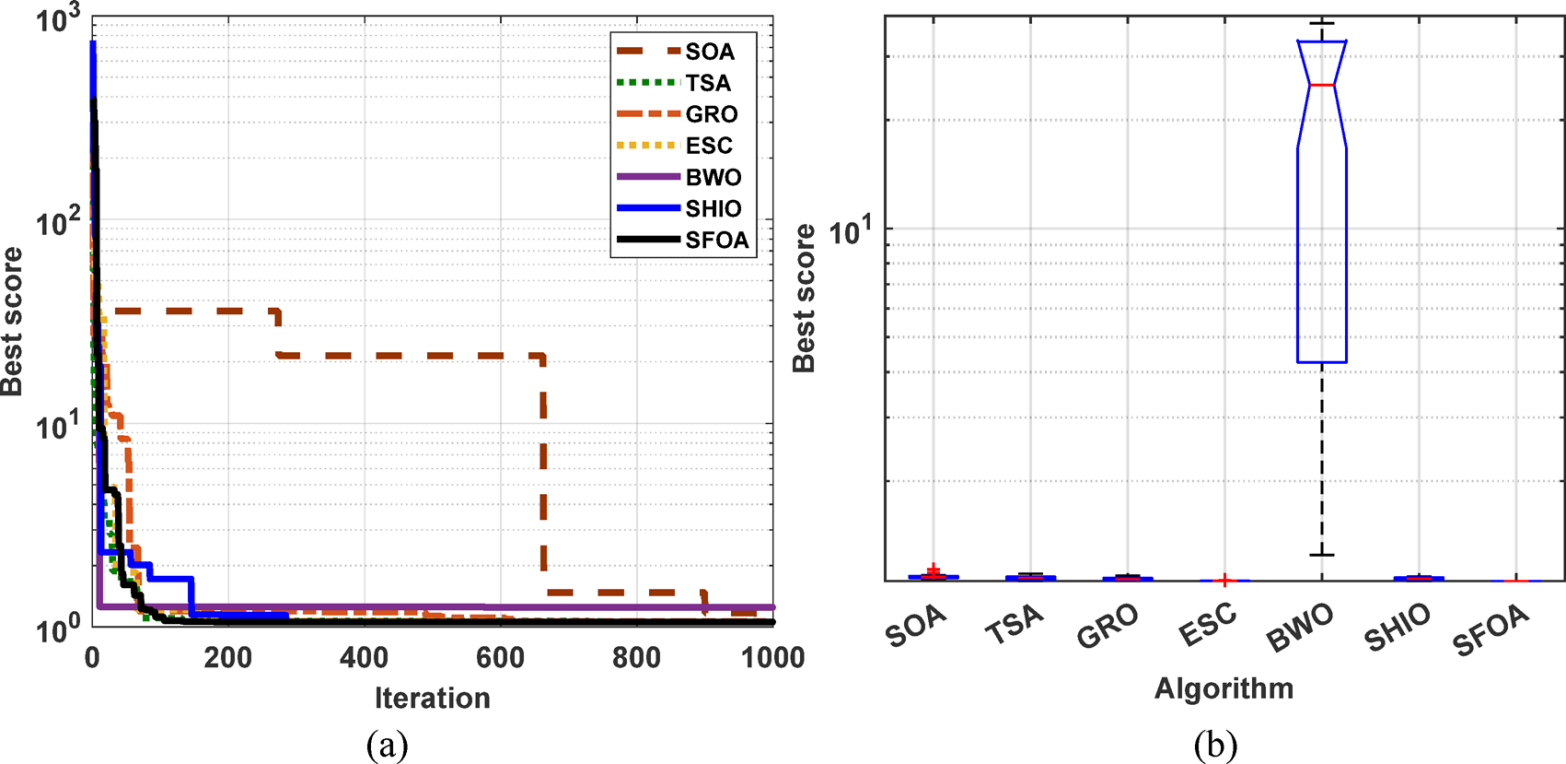
Variations of (**a**) best score versus iteration number, (**b**) boxplots for approaches applied on SR-12PEM 500-W.

The fourth FC studied in this work is SR-12PEM 500-W, the suggested SFOA, along with various optimization methods, is investigated to select the optimal parameters of the SR-12PEM 500 W. The optimal parameters and the SSE outcomes are displayed in Table 11. On the other side, Table 12 lists the worst, mean, best, and Std results from the optimization process. The acquired data revealed that the recommended SFOA outperformed all other approaches in obtaining the minimal SSE of 1. 056,370. On the other side, MHHO^37^ is the weakest strategy with SSE of 1.333. Figure 14 depicts the efficiency of all techniques during the procedure of iteration as well as boxplots for each. The plots verified the superior effectiveness of the suggested SFOA since it obtained the best fitness value quicker than the others and has the narrowest boxplot, ensuring satisfactory results while solving the issue.

**Table 11:** The optimized parameters SR-12PEM 500-W acquired through the suggested SFOA and other optimization techniques.

	*ζ*_*1*_	*ζ*_*2*_	*ζ*_*3*_	*ζ*_*4*_	*λ*	*R*_*c*_	*b*	*SSE*
SFOA	− 4.6126E−01	1.4937E−03	6.0721E−05	− 9.5400E−05	24.000	6.8288E−04	1.7538E−01	**1.056370E+00**
SOA	− 7.5072E−01	2.5961E−03	7.3330E−05	− 9.5400E−05	16.793	7.6705E−04	1.7132E−01	1.068728E+00
TSA	− 3.4818E−01	1.6219E−03	9.0467E−05	− 9.5400E−05	23.447	6.7013E−04	1.7551E−01	1.056532E+00
GRO	− 7.8298E−01	2.4228E−03	5.6520E−05	− 9.6204E−05	20.841	6.2459E−04	1.7508E−01	1.059956E+00
ESC	− 1.4001E+0	5.0000E−03	9.8000E−05	− 9.5400E−05	24.000	6.9272E−04	1.7519E−01	1.056394E+00
BWO	− 7.6838E−01	2.0461E−03	3.6000E−05	− 9.5400E−05	10.000	2.0150E−04	1.7334E−01	1.247798E+00
SHIO	− 1.3937E+0	3.9843E−03	3.6052E−05	− 9.5637E−05	19.817	6.3117E−04	1.7504E−01	1.058398E+00
CNRBO3^60^	− 9.7651E−01	2.8552E−03	4.8072E−05	− 9.5400E−05	23.9989	6.8059E−04	1.7541E−01	1.056500E+00
FMHHO^37^	− 8.5320E−01	3.0000E−03	7.8169E−05	− 9.5400E−05	23.000	1.0201E−04	1.5222E−01	1.251100E+00
MHHO^37^	− 8.5320E−01	3.3210E−03	9.8000E−05	− 9.5400E−05	23.000	2.6606E−04	1.4882E−01	1.333000E+00
VSDE^27^	− 8.5760E−01	3.0100E−03	7.7800E−05	− 9.5400E−05	23.000	1.3390E−04	1.5160E−01	1.266600E+00

Significant values are in [bold].

**Table 12:** Statistical indicators of different optimizers applied on SR-12PEM 500-W.

	Worst	Ave	Best	Std
SFOA	**1.056370E+00**	**1.056370E+00**	**1.056370E+00**	**4.514363E**−**15**
SOA	1.140293E+00	1.087460E+00	1.068728E+00	1.631409E−02
TSA	1.107059E+00	1.078224E+00	1.056532E+00	1.309502E−02
GRO	1.093977E+00	1.070982E+00	1.059956E+00	8.161049E−03
ESC	1.063133E+00	1.058092E+00	1.056394E+00	1.572765E−03
BWO	3.705474E+01	2.063625E+01	1.247798E+00	1.381601E+01
SHIO	1.085913E+00	1.072342E+00	1.058398E+00	8.363577E−03

Significant values are in [bold].

**Fig. 15:**
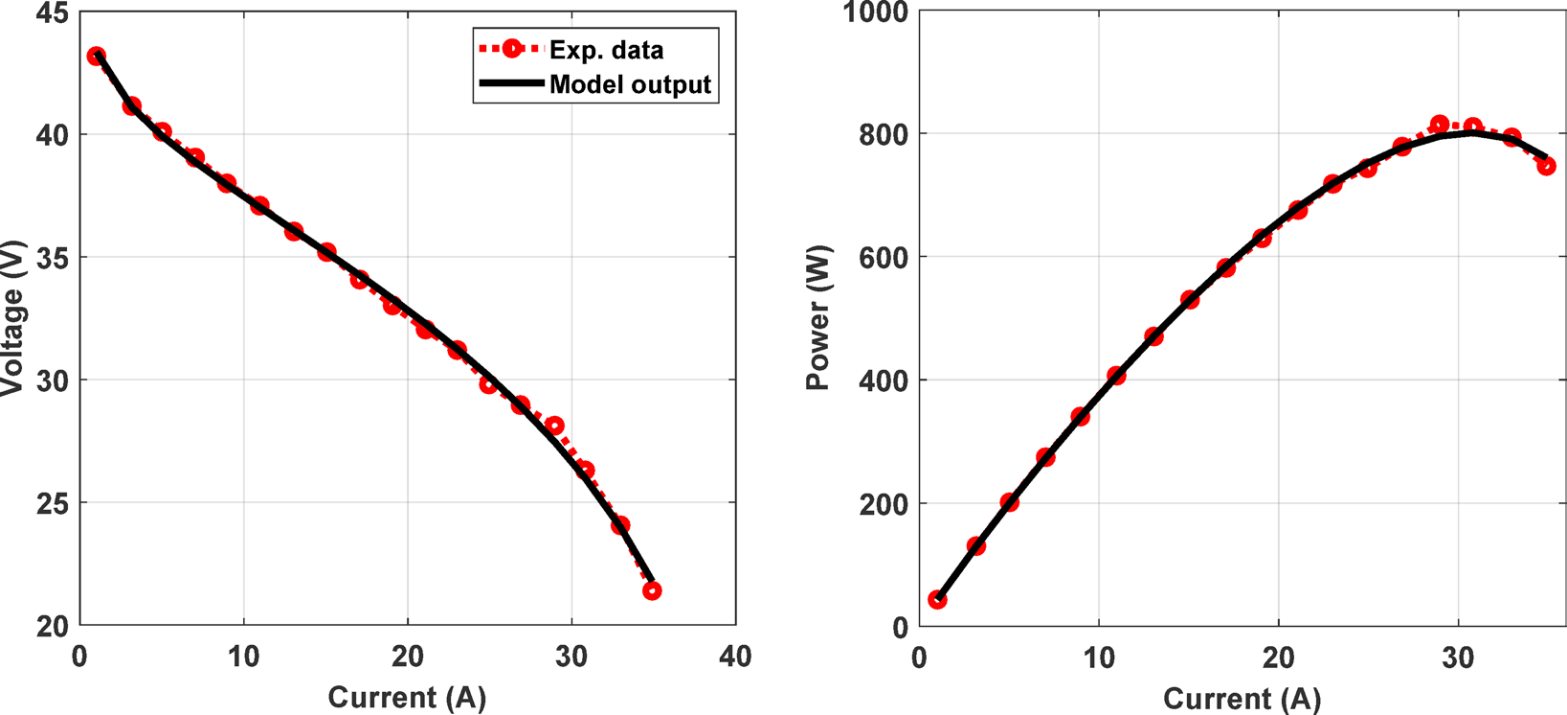
The I–V and I–P polarization curves of SR-12PEM 500-W.

The I-V and I-P polarization curves of the simulated and experimental data resulted from SFOA algorithm are shown in Fig. 15. The outcomes proved high match between the simulated and experimental data. The suggested approach is successful in establishing dependable equivalent circuit for the SR-12PEM 500-W.

### BCS-500W PEMFC stack

**Fig. 16:**
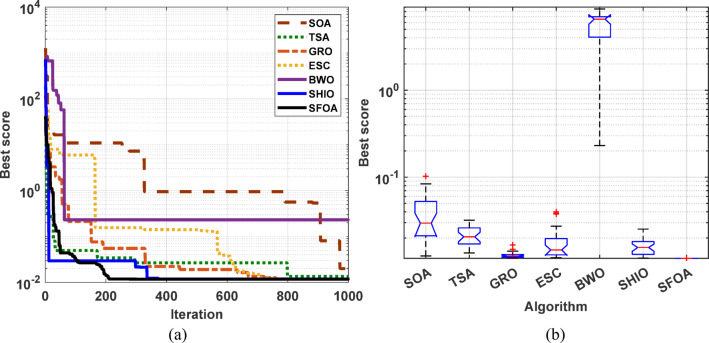
Variations of (**a**) best score versus iteration number, (**b**) boxplots for approaches applied on BCS-500W PEMFC.

**Fig. 17:**
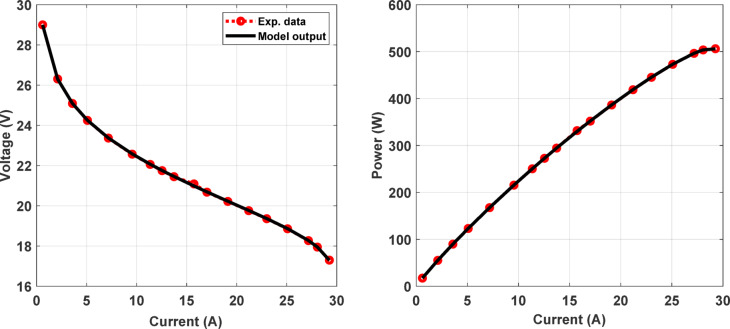
(**a**) I–V and (**b**) I–P polarization curves of BCS-500W PEMFC.

The fifth FC considered in this study is the BCS-500W PEMFC stack. The investigation experimentation of the BCS-500W PEMFC stack includes 32 fuel cells connected in series. The chief arrangements of the 500 W BCS stack can be observed in^37^. The existing of this FC should not exceed 30 A for thermal considerations. The optimum variables for the BCS-500 W FC are derived using the recommended SFOA and additional approaches and tabulated in Table 13. The highest achievable SSE is 1.169778E-02 through the suggested SFOA, whereas the lowest achievable is 2.309530E-01, achieved with BWO. Additionally, the statistical analyses are listed in Table 14. The SFOA achieved superior to all other techniques. Furthermore, Fig. 16a shows how the best score varies during the iterative process. The suggested technique outperforms the others in terms of reaction time, as it gets the optimum score after 210 iterations. Additionally, Fig. 16b shows the top scores and average rank for each optimizer. It is critical to note the rate at which convergence occurs between the experimental and evaluated curves of polarization generated via the suggested SFOA, as illustrated in Fig. 17, which verifies the suggested methodology’s outstanding effectiveness in building a reliable circuit for the BCS-500 W FC.

**Table 13:** The optimum variables for the BCS-500 W FC were derived using the recommended SFOA and additional approaches.

Optimizer	*ζ*_*1*_	*ζ*_*2*_	*ζ*_*3*_	*ζ*_*4*_	*λ*	*R*_*c*_	*b*	*SSE*
SFOA	− 8.7006E−01	2.5906E−03	5.7049E−05	− 1.9302E−04	20.877	1.0000E−04	1.6126E−02	**1.169778E−02**
SOA	− 8.5320E−01	3.1021E−03	9.1894E−05	− 1.9416E−04	23.000	2.2386E−04	1.6399E−02	1.242084E−02
TSA	− 9.7848E−01	3.0509E−03	6.5448E−05	− 1.9303E−04	23.000	2.3762E−04	1.6762E−02	1.346390E−02
GRO	− 9.5098E−01	3.0920E−03	7.3102E−05	− 1.9281E−04	21.679	1.7591E−04	1.6178E−02	1.177446E−02
ESC	− 1.0348E + 00	3.6557E−03	9.2465E−05	− 1.9253E−04	21.979	2.2503E−04	1.6098E−02	1.184078E−02
BWO	− 8.5320E−01	3.2194E−03	9.8000E−05	− 2.0916E−04	23.000	1.0000E−04	1.3600E−02	2.309530E−01
SHIO	− 1.1614E + 00	3.3256E−03	4.8369E−05	− 1.9278E−04	20.760	1.1185E−04	1.5998E−02	1.176097E−02
CMOA^38^	− 8.5321E−01	3.1410E−03	9.8000E−05	− 1.9299E−04	20.860	1.0000E−04	1.1612E−02	1.1698E−02
MOA^38^	− 1.1179E + 00	3.7185E−03	9.8000E−05	− 1.9107E−04	22.998	3.9573E−04	1.1581E−02	1.1290E−02
FMHHO^37^	− 8.7884E−01	3.0236E − 03	8.2272E − 05	− 1.9134E−04	22.709	4.0472E − 04	1.5289E − 02	1.1770E − 02
MHHO^37^	− 9.1048E − 01	3.0661E − 03	7.9053e − 05	− 1.9098E−04	19.384	1.0320E − 04	1.5212E − 02	1.3511E − 02
VSDE^27^	− 1.1970E + 00	4.2330E − 03	9.7990E − 05	− 1.9201E−05	20.194	1.108e − 04	1.57e − 02	1.21400E−02

Significant values are in [bold].

**Table 14:** Statistical indicators of different optimizers applied on BCS-500W PEMFC.

Optimizer	Worst	Ave	Best	Std
SFOA	**1.169778E−02**	**1.169778E−02**	**1.169778E−02**	**3.357672E−16**
SOA	1.025179E−01	3.789467E−02	1.242084E−02	2.285138E−02
TSA	3.207619E−02	2.135006E−02	1.346390E−02	5.317202E−03
GRO	1.662468E−02	1.271177E−02	1.177446E−02	1.042891E−03
ESC	4.004739E−02	1.805795E−02	1.184078E−02	8.041476E−03
BWO	8.583596E+00	5.408644E+00	2.309530E−01	2.357977E+00
SHIO	2.532047E−02	1.620514E−02	1.176097E−02	3.746182E−03

Significant values are in [bold].

Although the suggested SFOA-based technique shows better dynamic performance and excellent accuracy. There is no real-time experimental implementation; instead, the validation is mostly based on simulation findings utilizing previously released datasets. The suggested model has been tested under different operating settings.

### Statistical analysis

**Fig. 18:**
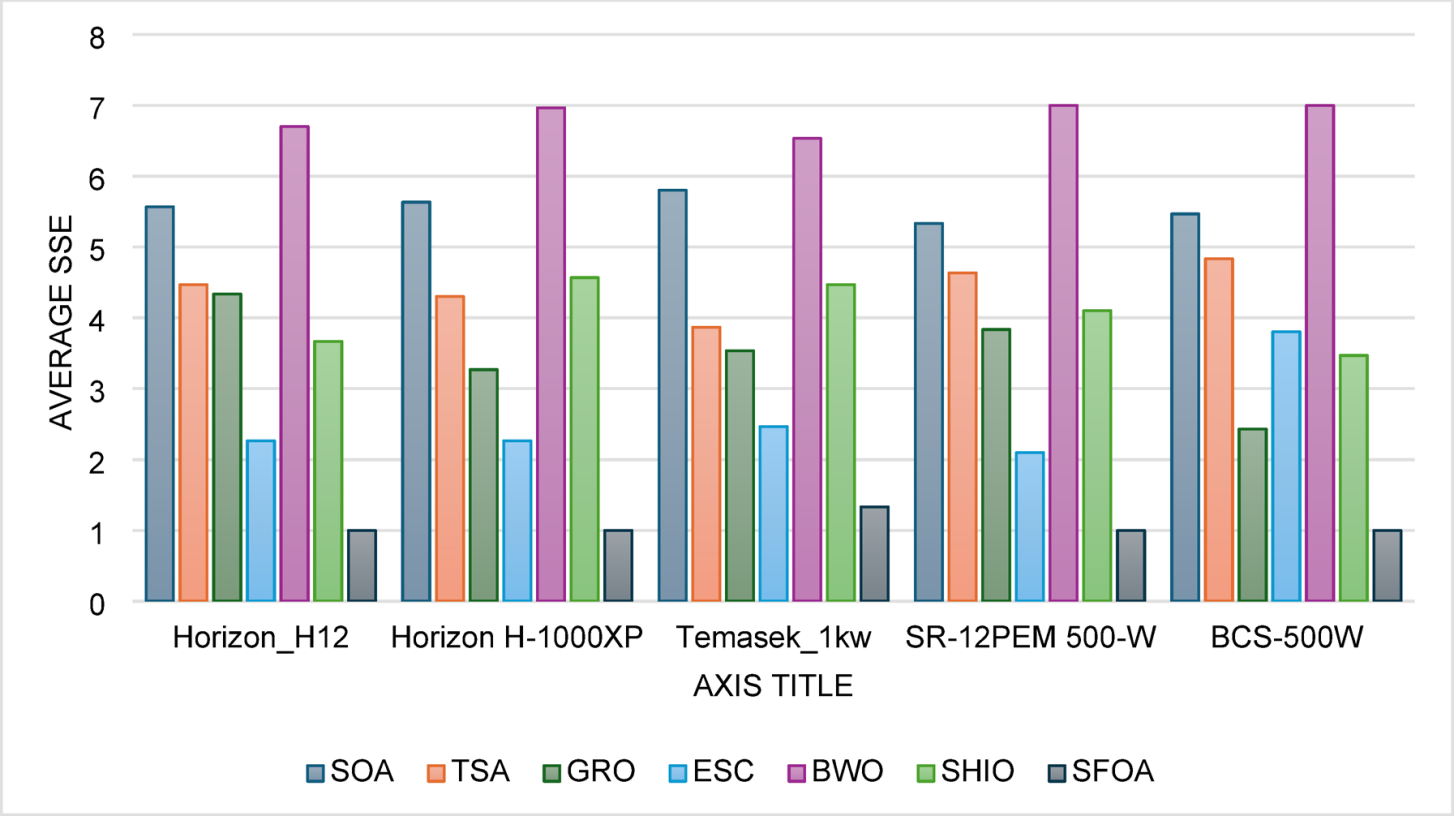
PEMFC’s average ranking SSE as determined by the Friedman test.

The suggested SFOA is tested using Friedman ANOVA and Kruskal–Wallis tests based on scores from multiple runs. Figure 18 depicts the average ranking of fitness values acquired by the Friedman test. The proposed SFOA clearly ranked first in all addressed FCs. Tables 15, 16, and 17 show the Friedman ANOVA findings, Kruskal–Wallis outcomes, and ANOVA outcomes for each FC. It should be observed that the p-value is lower than 0.05 in each case, confirming the effectiveness of the suggested SFOA in obtaining the parameters of the fuel cell.

**Table 15:** Friedman ANOVA results.

PEMFC	Source	SS	df	MS	Chi-sq	Prob > Chi-sq
Horizon H-12 PEMFC	Columns	665.6667	6	110.9444	142.6429	2.77E−28
	Error	174.3333	174	1.001916		
	Total	840	209			
Horizon H-1000XP PEMFC stack	Columns	732.6667	6	122.1111	157	2.56E−31
	Error	107.3333	174	0.616858		
	Total	840	209			
Temasek_1kw	Columns	587.2	6	97.86667	125.8286	9.70E−25
	Error	252.8	174	1.452874		
	Total	840	209			
SR-12PEM 500-W	Columns	714.8	6	119.1333	153.1714	1.65E−30
	Error	125.2	174	0.71954		
	Total	840	209			
BCS-500W PEMFC stack	Columns	708.7333	6	118.1222	151.8714	3.11E−30
	Error	131.2667	174	0.754406		
	Total	840	209

**Table 16:** Kruskal–Wallis results.

PEMFC	Source	SS	df	MS	Chi-sq	Prob > Chi-sq
Horizon H-12 PEMFC	Columns	612,089.1	6	102,014.8	165.7655	3.56E−33
	Error	159,643.4	203	786.4209		
	Total	771,732.5	209			
Horizon H-1000XP PEMFC stack	Columns	647,007.1	6	107,834.5	175.222	3.51E−35
	Error	124,725.4	203	614.411		
	Total	771,732.5	209			
Temasek_1kw	Columns	536,947	6	89,491.17	145.4175	7.20E−29
	Error	234,775.5	203	1156.53		
	Total	771,722.5	209			
SR-12PEM 500-W	Columns	646,024.9	6	107,670.8	174.9563	3.99E−35
	Error	125,706.1	203	619.242		
	Total	771,731	209			
BCS-500W PEMFC stack	Columns	629,739.2	6	104,956.5	170.5455	3.45E−34
	Error	141,993.3	203	699.4744		
	Total	771,732.5	209

**Table 17:** The ANOVA result.

PEMFC	Source	SS	df	MS	F	Prob > F
Horizon H-12 PEMFC	Columns	0.001334	6	0.000222	4.74163	1.52E−04
	Error	0.009517	203	4.69E−05		
	Total	0.010851	209			
Horizon H-1000XP PEMFC stack	Columns	56.77101	6	9.461835	27.98749	2.98E−24
	Error	68.62897	203	0.338074		
	Total	125.4	209			
Temasek_1kw	Columns	0.503938	6	0.08399	32.48472	2.80E−27
	Error	0.52486	203	0.002586		
	Total	1.028798	209			
SR-12PEM 500-W	Columns	9843.845	6	1640.641	60.16518	1.99E−42
	Error	5535.595	203	27.26894		
	Total	15,379.44	209			
BCS-500W PEMFC stack	Columns	746.7881	6	124.4647	156.6808	2.36E−73
	Error	161.2599	203	0.794384		
	Total	908.048	209			

The obtained findings verified that, the recommended method is preferred for constructing a trustworthy model of various PEMFCs by determining their ideal parameters.

## Conclusion

To identify the ideal parameters of several PEMFCs operating in distinct situations, this study suggests a novel method using the starfish optimizer algorithm (SFOA). Seven factors of ζ_1_, ζ_2_, ζ_3_, ζ_4_, λ, R_c_, and b are the design variables to be determined, while the target is to reduce the SSE between the measured and calculated data. Five distinct FCs are analyzed: Horizon_H12, Horizon H-1000XP PEMFC stack, Temasek_1kw, SR-12PEM 500-W, and BCS-500W PEMFC stacks. The fetched outcomes via the suggested SFOA are compared with many other algorithms such as SOA, TSA, GRO, ESC, BWO, SHIO, CNRBO3, FMHHO, MHHO, and VSDE.

The findings obtained are listed as follows:

- The suggested SFOA acquired the lowest SSE of 4.662696E-04 for Horizon H-12, while the worst SSE of 1.160000E-01 is obtained using the reported WOA^74^.
- Regarding the Horizon H-1000XP FCs, the SFOA attained the lowest target value of 3.948527E-01, while the worst value is 4.588490E-01 through BWO.
- The minimum SSE achieved for Temasek_1kw is 5.4322160E-01 by the suggested SFOA, but the worst one is 7.986870E-01 by Rao-3^62^.
- In SR-12 500 W and BCS-500 W FCs, the worst target values are 1.333000E+00 and 2.309530E-01obtained via MHHO^37^ and BWO, respectively, while the best target values of 1.056370E+00 and 1.169778E-02 are obtained via SFOA.

Additionally, the established models are examined dynamically by building the model in Simulink/Matlab while step load disruption is applied. It is noticed that the voltage at the terminal in accordance matches the load disruption, confirming the effectiveness of established model via SFOA. In future work, the FC-established models will be integrated into energy management systems, and their performances will be analyzed. Moreover, setup for experiments will be conducted in the next work to store accurate measured data of the curves of polarization for various FCs.

## Data Availability

All data generated or analysed during this study are included in this published article.
